# Identification of a Pentasaccharide Lead Compound with High Affinity to the SARS-CoV-2 Spike Protein via In Silico Screening

**DOI:** 10.3390/ijms242216115

**Published:** 2023-11-09

**Authors:** Binjie Li, Tianji Zhang, Hui Cao, Vito Ferro, Jinping Li, Mingjia Yu

**Affiliations:** 1 Beijing Advanced Innovation Center for Soft Matter Science and Engineering, Beijing University of Chemical Technology, Beijing 100029, China; 2023400367@buct.edu.cn; 2 Division of Chemistry and Analytical Science, National Institute of Metrology, Beijing 100029, China; zhangtianji@nim.ac.cn; 3 College of Life Science and Technology, Beijing University of Chemical Technology, Beijing 100029, China; caohui@mail.buct.edu.cn; 4 School of Chemistry & Molecular Biosciences, The University of Queensland, Brisbane 4072, Australia; v.ferro@uq.edu.au; 5 Department of Medical Biochemistry and Microbiology, Uppsala University, 752 36 Uppsala, Sweden; 6 School of Chemistry and Chemical Engineering, Beijing Institute of Technology, Beijing 100081, China

**Keywords:** SARS-CoV-2, RBD, heparan sulfate, protein binding, virtual screening

## Abstract

The spike (S) protein on the surface of the SARS-CoV-2 virus is critical to mediate fusion with the host cell membrane through interaction with angiotensin-converting enzyme 2 (ACE2). Additionally, heparan sulfate (HS) on the host cell surface acts as an attachment factor to facilitate the binding of the S receptor binding domain (RBD) to the ACE2 receptor. Aiming at interfering with the HS-RBD interaction to protect against SARS-CoV-2 infection, we have established a pentasaccharide library composed of 14,112 compounds covering the possible sulfate substitutions on the three sugar units (GlcA, IdoA, and GlcN) of HS. The library was used for virtual screening against RBD domains of SARS-CoV-2. Molecular modeling was carried out to evaluate the potential antiviral properties of the top-hit pentasaccharide focusing on the interactive regions around the interface of RBD-HS-ACE2. The lead pentasaccharide with the highest affinity for RBD was analyzed via drug-likeness calculations, showing better predicted druggable profiles than those currently reported for RBD-binding HS mimetics. The results provide significant information for the development of HS-mimetics as anti-SARS-CoV-2 agents.

## 1. Introduction

Severe acute respiratory syndrome coronavirus 2 (SARS-CoV-2) is the causative agent of coronavirus disease 2019 (COVID-19), which was declared a global pandemic by the World Health Organization (WHO) in 2020. Although the pandemic situation is under control at present, whether new variants will emerge is unpredictable, thus COVID-19 still poses a great threat to human health [1]. SARS-CoV-2 is a member of the betacoronaviruses [2], and infects human cells via a trimeric S (spike) protein on its surface binding to the angiotensin-converting enzyme 2 (ACE2) receptor [3] through the receptor-binding domain (RBD) in an ‘open’ conformation [4]. Heparan sulfate (HS) has been identified [5] as a coreceptor priming the S protein for ACE2 interaction [6], which then triggers the fusion of the viral membrane with the host cell membrane [7]. Mutations within the S protein of SARS-CoV-2 emerged rapidly, which is assumed to alter virus–host cell interactions [8,9,10]. Vaccines are critical tools to reduce the severity of virus-induced diseases; however, vaccine effectiveness against SARS-CoV-2 is threatened by the emergence of viral variants [11]. Therefore, it is necessary to develop drugs targeting viral infection, including the inhibition of viral entry, virus replication and release, and virus-induced inflammation. Since HS plays important roles in virus–cell receptor interactions, identifying HS-mimetics as potential inhibitors for targeting S protein-HS-ACE2 interactions is a valid approach to attenuate SARS-CoV-2 infection [12,13,14]. Here, we have established an unprecedented pentasaccharide library containing 14,112 species. Through virtual screening of the binding between RBD and the pentasaccharides, we have identified a potent lead structure with a high affinity for the RBD. Drug-likeness calculations showed better the properties of the pentasaccharide than those of the reported oligosaccharide compounds. Collectively, our results provide insights into the potential to develop HS-mimetics as therapeutic agents which prevent SARS-CoV-2 binding to target cells.

## 2. Results

### 2.1. Identification of the RBD-Binding Pentasaccharide

**Figure 1:**
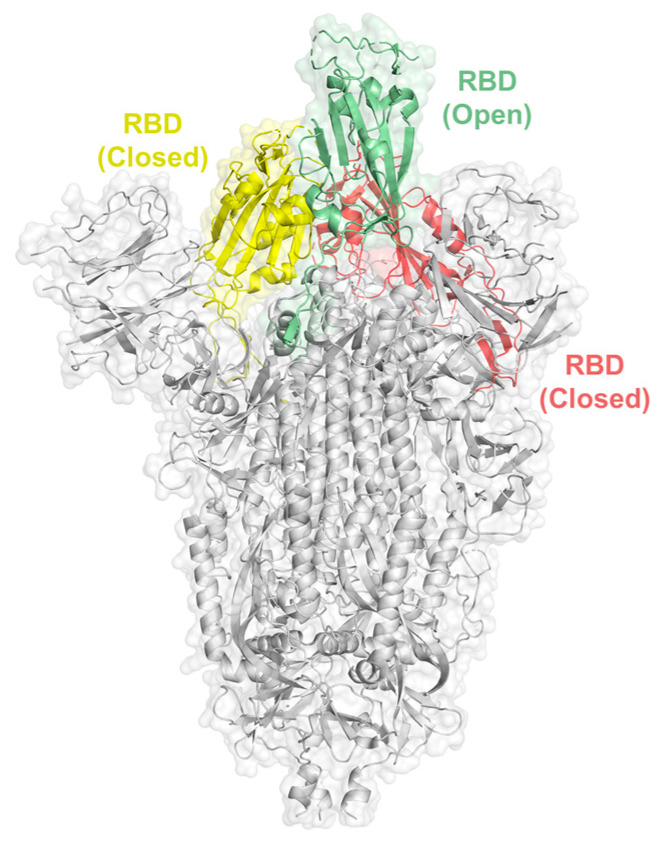
Structure of the trimeric SARS-CoV-2 S protein showing the open and closed form of RBD (PDB ID: 6VSB) [19].

**Figure 2:**
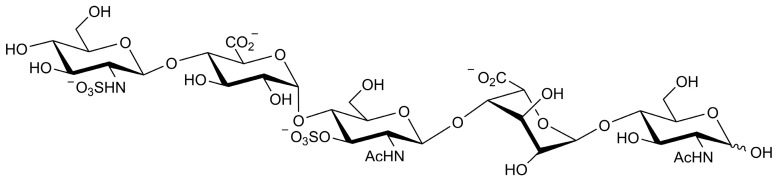
Structure of the top-hit pentasaccharide AD08043.

The trimeric SARS-CoV-2 S protein engages human ACE2 with a single RBD extending from the trimer in an “open” active conformation (Figure 1) and mediates the entry of virions into target cells [15]. There is a cluster of positively charged amino acid residues adjacent to the ACE2 binding site in the open RBD conformation, serving as a binding site for HS [14]. To identify oligosaccharides (HS-mimetics) binding to the RBD with high affinity, we established an unprecedented virtual library of pentasaccharides, using the backbone sequences of five sugar units of glucuronic acid, iduronic acid, and glucosamine, with sulfate substitution in the possible positions, resulting in a library of 14,112 different structures. The pentasaccharide library was virtually screened for a binding to the S protein with open RBD conformation, and the top-hit pentasaccharide (designated AD08043, Figure 2) is GlcNS-β-(1→4)-GlcA-α-(1→4)-GlcNAc3S-β-(1→4)-IdoA-α-(1→4)-GlcNAc. This pentasaccharide contains one IdoA unit that increases its conformational flexibility [16], and a 3-*O*-sulfated glucosamine unit that is a signature of the antithrombin binding epitope of heparin and the herpes virus binding epitope of HS [17,18]. Moreover, we found that the top ten pentasaccharides virtually screened, including AD08043, all exhibited a poor degree of sulfation (Table S1).

### 2.2. Analysis of the Molecular Interactions via Molecular Modelling

**Figure 3:**
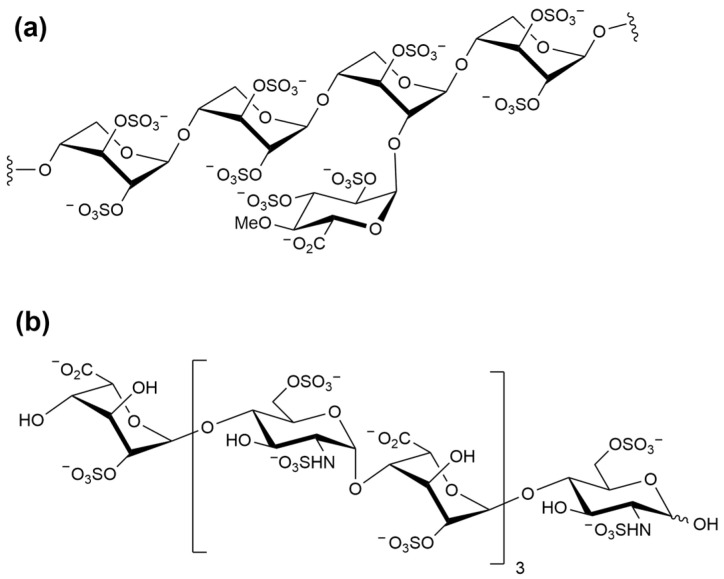
Structure of (**a**) pentosan polysulfate (PPS) [20]; (**b**) (IdoA2S-GlcNS6S)_4_ [6].

Next, we analyzed binding affinity of the selected pentasaccharide AD08043 with RBD in comparison with other oligosaccharides that have shown a binding to RBD (Figure 3), including the pentosan polysulfate monomer (PPS) [20,21] and (IdoA2S-GlcNS6S)_4_ [6] via the molecular docking of SARS-CoV-2 RBD (Table 1). The results indicate that AD08043 binds more tightly with SARS-CoV-2 RBD (with a binding energy of −7.149 kcal/mol) than (IdoA2S-GlcNS6S)_4_ and PPS (with binding energies of −6.797 and −6.247 kcal/mol, respectively).

**Figure 4:**
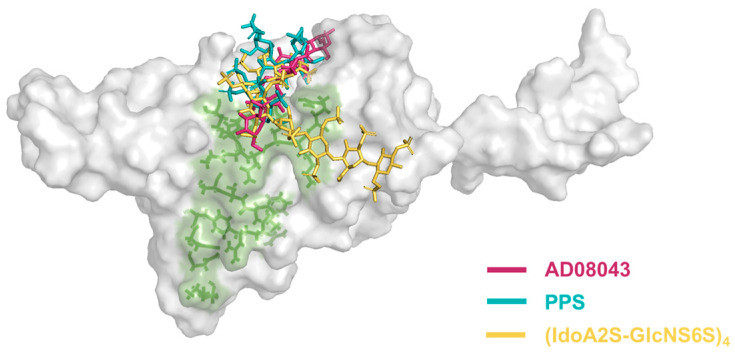
The binding molecular model of oligosaccharides of the HS-analogue and SARS-CoV-2 RBD. The green surface indicates the RBD-ACE2-RBD binding interface. AD08043 is colored in purple, PPS is colored in teal, and (IdoA2S-GlcNS6S)_4_ is colored in yellow.

To generate further insights into the intermolecular interactions and stability of the complexes between the pentasaccharide and RBD protein, we examined the weak non-bond forces such as hydrogen bonds and hydrophobic contacts for each of the amino acids involved in the binding (Table 2). Hydrogen bonding has a diverse role in ligand–receptor interactions. The favorability of the ligand–receptor hydrogen bonds depends upon the total energy change involved in the formation and breaking of all these hydrogen bonds. Likewise, the ligand–receptor hydrogen bonds are considered key contributors to binding due to their specificity. AD08043 shows seven hydrogen bonds involving amino acid residues of Arg355 (3.18 Å and 3.06 Å), Arg466 (3.14 Å, 3.11 Å, and 3.18 Å), Tyr396 (2.94 Å and 3.14 Å), and Leu517 (3.11 Å). In comparison, the (IdoA2S-GlcNS6S)_4_ oligosaccharide shows a maximum of 24 interactions, including thirteen hydrogen bonds with binding interface amino acid residues Arg355 (3.11 Å), Arg357 (3.19 Å, 2.95 Å, 3.06 Å, 3.00 Å, 2.96 Å, and 3.24 Å), and Arg466 (3.14 Å and 3.18 Å), as well as eleven hydrophobic contacts with the binding interface amino acid residues Trp353 and Asn354. PPS shows six hydrogen bonds with amino acid residues of Arg355 (2.93 Å and 3.14 Å), Arg357 (3.03 Å and 3.35 Å), and Arg466 (3.28 Å and 3.03 Å) (all of which are located in the binding interface), and eleven hydrophobic contacts. The results of the molecular modeling also showed that AD08043 was located on the binding interface of RBD and ACE2 (Figure 4).

Interestingly, although the number and length of the hydrogen bonds of AD08043 is less than PPS and (IdoA2S-GlcNS6S)_4_, the binding affinity of AD08043 to RBD is stronger. This may be ascribed to the 3-*O*-sulfation substitution.

**Figure 5:**
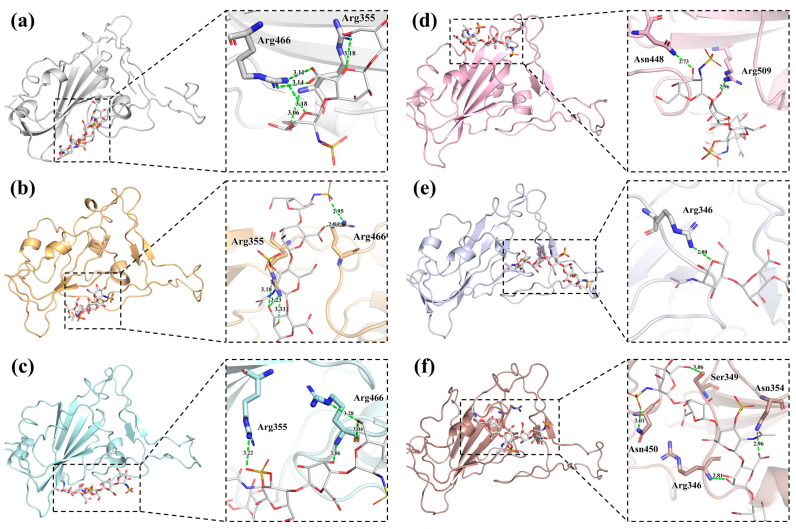
Interactions between the interface amino acids of the RBDs of the variants of SARS-CoV-2 and AD08043. (**a**) Original variant; (**b**) B.1.1.7 (alpha) variant; (**c**) B.1.351 (beta) variant; (**d**) P.1(gamma) variant; (**e**) B.1.617.2 (delta) variant; and (**f**) B.1.1.529 (omicron BA.1) variant.

Furthermore, the binding affinity of AD08043 with the RBD of several SARS-CoV-2 variants was also analyzed (Table S2). The results demonstrated a high-affinity binding of AD08043 to the RBD of SARS-CoV-2 variants (Table 3). During the interaction with AD08043, the SARS-CoV-2 B.1.1.7 (alpha) variant’s RBD shows six hydrogen bonds with the binding interface amino acid residues of Arg355 (3.18 Å, 3.33 Å, and 3.23 Å) and Arg466 (2.84 Å and 2.95 Å) as well as nine hydrophobic contacts. The SARS-CoV-2 B.1.351 (beta) variant’s RBD shows six hydrogen bonds with the binding interface amino acid residues Arg355 (3.22 Å) and Arg466 (3.28 Å and 3.06 Å), as well as seven hydrophobic contacts. The SARS-CoV-2 P.1(gamma) variant’s RBD shows two hydrogen bonds with the binding interface amino acid residues Asn448 (2.73 Å) and Arg509 (2.96 Å), as well as eleven hydrophobic contacts with the binding interface amino acid residues Arg346, Asn450, and Tyr451. The SARS-CoV-2 B.1.617.2 (delta) variant’s RBD shows six hydrogen bonds with the binding interface amino acid residues Arg346 (2.80 Å) as well as nine hydrophobic contacts with the binding interface amino acid residues Ala348, Ala352, Asn354, and Tyr451. The SARS-CoV-2 B.1.1.529 (omicron BA.1) variant’s RBD shows six hydrogen bonds with the binding interface amino acid residues Arg346 (2.81 Å), Ser349 (3.06 Å), Asn354 (2.96 Å), and Asn450 (3.01 Å), as well as eight hydrophobic contacts with the binding interface amino acid residues Phe347, Ala348, and Ala352. Exceptionally, the B.1.351 (beta) variant’s RBD has shorter hydrogen bond lengths and more hydrophobic contacts. It should be pointed out that the interactions between AD08043 and the RBDs of the different variants of SARS-CoV-2 are located around the interface of the RBD-ACE2 (Figure 5). To provide a more comprehensive analysis, we further analyzed the binding affinity and intermolecular interactions of the top ten pentasaccharides with the RBD. The results demonstrated that the other pentasaccharides bind with a slightly lower affinity than AD08043. Additionally, the top ten pentasaccharides all bind to the RBD at the RBD-HS-ACE2 binding interface, demonstrating a comparable binding pattern with the AD08043-RBD complex (Figure S1 and Table S3).

**Figure 6:**
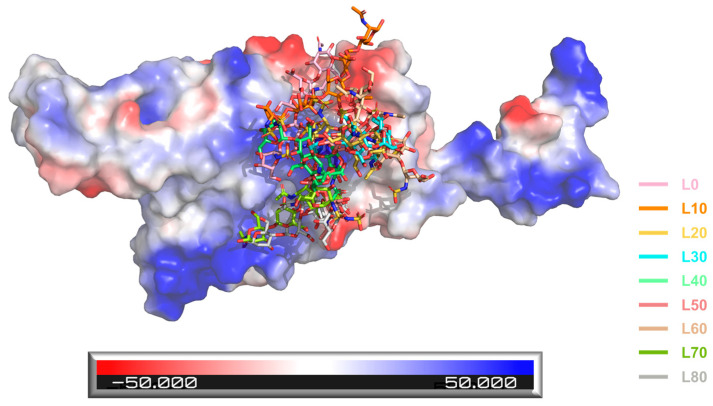
Electrostatic surface rendering of the SARS-CoV-2 RBD-AD08043 complex during the MD simulation. Blue and red surfaces indicate electropositive and electronegative surfaces, respectively. L0, L10, L20, L30, L40, L50, L60, L70, and L80 are colored in pink, orange, yellow, cyan, pale green, salmon, wheat, limon, and gray (e.g., L10 represents the location of AD08043 at 10 ns during the MD simulation period).

Next, we conducted MD simulations to verify the reliability of the model constructed from docking. Quantitatively, the RMSD values of the structures from the MD and docking are small (Figure S2), which indicates that the structural variation is minor between the MD simulations and docking. Furthermore, to explore the SARS-CoV-2 RBD-AD08043 binding mechanism during conformation changes, we intercepted a conformation every 10 ns and calculated the electrostatic potential map of the RBD. The results revealed an extended electropositive surface with dimensions and turns/loops consistent with an HS-binding site identified earlier [4,14] (Figure 6), e.g., around the binding interface between RBD and ACE2. AD08043, in almost all of the different time points (except for 10 ns, colored orange in Figure 6), was located around the RBD electropositive surfaces.

To understand the molecular interaction mechanisms, configurations of the SARS-CoV-2 RBD-AD08043 complex were analyzed every 10 ns during the MD simulation (80 ns) (Table 4). The molecular interactions shown are those mediated by hydrogen bonds and hydrophobic contacts that play a major role in forming the SARS-CoV-2 RBD-AD08043 complexes. At 0 ns of the MD simulation, AD08043 shows a maximum of 13 interactions including five hydrogen bonds with the binding interface amino acid residues Arg355 (3.07 Å and 2.65 Å) and Arg466 (2.77 Å and 2.83 Å), as well as eight hydrophobic contacts with the binding interface amino acid residues Trp353. At 10 ns, AD08043 shows two hydrogen bonds due to the initial unstable stage of the MD simulation which is consistent with the electrostatic convergence interaction result when AD08043 is positioned on the electronegative surface of the RBD. At 20 ns, AD08043 shows five hydrogen bonds with the binding interface amino acid residues Arg357 (2.60 Å, 2.81 Å, and 2.88 Å). At 30 ns, AD08043 shows four hydrogen bonds with the binding interface amino acid residues Arg357 (2.67 Å and 2.76 Å). However, there is no non-bond interaction at 40 ns, showing that the complex is in a relatively unstable state, which is consistent with the RMSD results, as the RMSD values from the MD and docked structures fluctuate considerably around this time point. At 50 ns, AD08043 shows one hydrogen bond as well as two hydrophobic contacts with the binding interface amino acid residues Arg355 and Arg357. At 60 ns, AD08043 shows five hydrogen bonds with the binding interface amino acid residues Arg357 (2.81 Å, 2.96 Å, and 3.28 Å) as well as two hydrophobic contacts. At 70 ns, AD08043 shows two hydrogen bonds with the binding interface amino acid residues Asn354 (3.10 Å) and Arg357 (3.04 Å), as well as eight hydrophobic contacts with the binding interface amino acid residues Arg346, Phe347, Ala348, Arg355, Lys356. At 80 ns, AD08043 shows three hydrogen bonds with the binding interface amino acid residues Arg346 (2.92 Å), Lys356 (3.03 Å), and Arg357 (2.97 Å), as well as two hydrophobic contacts with the binding interface amino acid residues Phe347 and Ala348.

**Figure 7:**
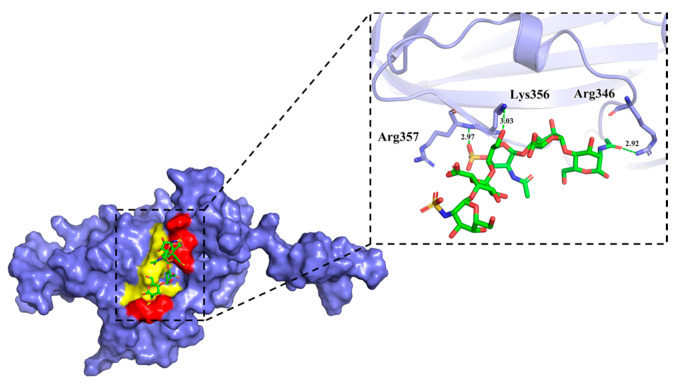
Detailed information on the interactions between SARS-CoV-2 RBD and AD08043 at 80 ns during the MD simulation period. Amino acids that produce polar and non-polar interactions with the ligand are colored in red and yellow, respectively.

Interestingly, at the late MD time points (70 ns and 80 ns), all RBD-HS-ACE2 interfacial amino acid residues are involved in non-bond interactions. Aside from the unstable time points of 10 ns and 40 ns, the measured dynamic interaction involved Arg357, which is likely a key amino acid for maintaining structural stability during the conformational changes of the complex. Notably, the interactions between AD08043 and SARS-CoV-2 RBD in the process of the MD simulation mainly involve the sulfate groups (3OS, NS), carboxyl group on the GlcA unit, and the hydroxyl groups on AD08043 skeletons (Figures S4–S12). Further, 3-*O*-sulfation on the GlcNAc unit seems to be critical for stabilization of the RBD-AD08043 complex, since it is directly involved in all the interactions during the entire period of MD analysis after stabilization. We have also noticed that the initial phase of the interaction involved amino acids both located at the interaction interface and outside of the interface; however, towards the end of the interaction, only interface amino acids are involved (Figure 7). By the end of the MD simulation, AD08043 is completely located on the RBD-HS-ACE2 binding interface. We also performed point mutations to re-assess the effects of key amino acids on the HS-RBD interaction. As expected, the point mutations of the amino acids involved in hydrogen bond formation, through their conversion to alanine residues, lead to the loss of RBD-HS-ACE2 binding interface interactions (Figure S13).

To confirm the affinity of oligosaccharides towards the RBDs of the variants of SARS-CoV-2 in the ligand–protein complexes, binding free energy calculations were carried out. The calculated results of the Poisson–Boltzmann surface area (MMPBSA) binding free energy are represented in Table S4. The overall trend of the binding free energy values of oligosaccharides–RBD complexes, in decreasing order, is given as PPS < (IdoA2S-GlcNS6S)_4_ < AD08043, respectively. The complexes AD08043 and RBD demonstrated the highest binding free energy values compared to other oligosaccharide complexes. Moreover, comparing the binding affinity of AD08043 with the RBD of the various SARS-CoV-2 variants under investigation revealed that the delta and omicron variants had a comparatively lower affinity, which is consistent with the docking results.

### 2.3. Frontier Molecular Orbital Analysis of the Ligands

**Figure 8:**
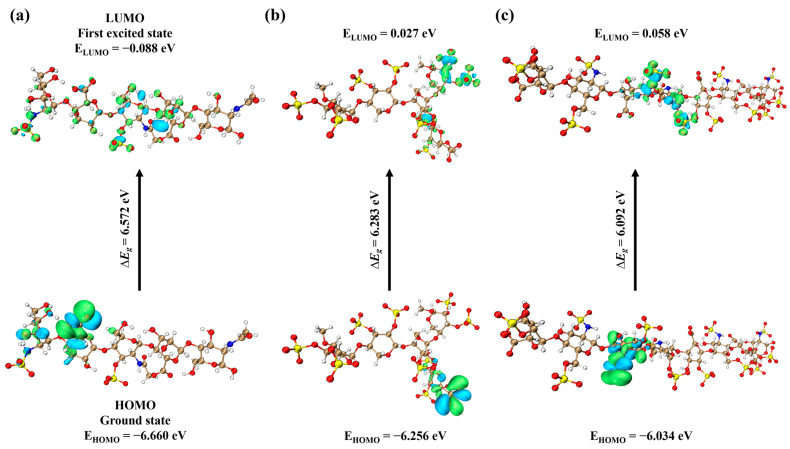
The FMOs including HOMO and LUMO for (**a**) AD08043; (**b**) PPS; and (**c**) (IdoA2S-GlcNS6S)_4_, as calculated at B3LYP-D3/6-31 + G(d,p) level of DFT.

Frontier molecular orbitals (FMOs) are usually referred to as the highest occupied molecular orbital (HOMO) and lowest unoccupied molecular orbital (LUMO). The structural properties of the ligands used for developing the docking interactions are important in explaining the density distribution pattern on frontier molecular orbitals [22]. The energetics of FMOs are crucial to the reactivities and chemical structures of ligand molecules. Differences in HOMO-LUMO energy is of importance in determining the reactivity and stability of the molecules. To explore the chemical stability of the pentasaccharide, we performed FMO analysis in comparison with the reported oligosaccharides. The energetic results show that the ∆*E_g_* values of the HOMO-LUMO gap of AD08043, PPS, and (IdoA2S-GlcNS6S)_4_ are 6.572, 6.283, and 6.092 eV, indicating that the structure of AD08043 is more stable (Figure 8 and Table 5).

### 2.4. Evaluation of the HS-Analogues for Druggability via ADMET Studies

Having observed the high affinity binding between AD08043 and RBD at the interaction interface of ACE2-RBD, it should be interesting to explore the feasibility of developing AD08043 into a therapeutic drug to prevent SARS-CoV-2 from binding to ACE2. Analysis via ADMET (Table 6) revealed that AD08043 possesses a high TPSA as well as aqueous solubility due to its polar groups. The parameter of the Plasma Protein Binding (PPB) being in the range of <90% indicates a relatively high therapeutic response [23]. The results also indicate that AD08043 has an appropriate in vivo volume distribution, falling within the range of 0.04–20 L/kg. Adhering to the Pfizer Rule [24], that substances with a low log P (<3) and high TPSA (>75) are likely to be non-toxic, AD08043 is unlikely to cause drug-induced toxicity, e.g., liver damage, acute oral toxicity, skin sensitization, carcinogenicity, and respiratory toxicity. Based on its pharmacokinetics and toxicity, AD08043 is predicted to be a drug-like chemical. At the same time, we also compared the HS-analogues of PPS and (IdoA2S-GlcNS6S)_4_. As shown in Table 6, AD08043 is superior to the other two structures in several parameters of druggability. Notably, all three structures displayed poor blood–brain barrier permeability, which may limit their penetration into the brain; however, none of the structures is predicted to impact the five major cytochrome P450 (CYP) isoforms, e.g., CYP1A2, CYP2C19, CYP2C9, CYP2D6, and CYP3A4.

## 3. Discussion

HS acts as a co-receptor priming SARS-CoV-2 RBD for ACE2 interaction, thus assisting the virus’ attachment and entry. Previous studies have shown that HS-mimetics display potent anti-SARS-CoV-2 activity, and the concept of interfering with HS–virus interactions using HS-mimetics for prophylactic as well as therapeutic purposes against virus is drawing attention [5,12,13,21,25]. Inspired by the previous findings, we employed a molecular modelling approach, aiming to find HS-mimetics binding to the SARS-CoV-2 RBD with high affinity. Virtual screening of an in-house built pentasaccharide library for their binding to the SARS-CoV-2 RBD identified one top-ranked structure, GlcNS-GlcA-GlcNAc3S-IdoA-GlcNAc, known as AD08043. This unique structure has a relatively low degree of sulfation, but displayed the highest binding affinity to the SARS-CoV-2 RBD, which reveals that the binding affinity is closely related not only to the number and site of negatively charged groups in the oligosaccharide sequence, but also to the changes in RBD protein conformation and the site of key basic amino acids during the interaction. Notably, the pentasaccharide has a 3-*O*-sulfate substitution that is present in the antithrombin-binding sequence of heparin and the herpes simplex virus binding domain of HS.

MD simulations were performed to determine the fluctuation and conformational changes of AD08043 binding to the RBD under physiological conditions. The result demonstrates that AD08043 at different time points (except for 10 ns) is located around the RBD electropositive surfaces, which is consistent with the mechanism of negatively charged HS-mimetics binding to positively charged amino acid patches in the target protein. In particular, Arg357 repeatedly occurred during this process, suggesting a key role of the amino acid in the conformational stability of the AD08043-SARS-CoV-2 RBD interaction. Additionally, the amino acid residues involved in each period of the MD simulation illustrate that there are non-interface amino acids that interact before the MD simulation (during docking), whereas the amino acid residues that interact at the end of the MD simulation (70 ns, 80 ns) all belong to the interface amino acids, and AD08043 is completely located on the RBD-ACE2 binding interface. This is consistent with the results obtained from our earlier study [26]. This finding may represent a general mechanism of oligosaccharides interacting with amino acids at the binding interface of the RBD.

Comparing the binding affinity and intermolecular interaction of AD08043 with the RBDs of several SARS-CoV-2 variants of concern revealed the relatively lower affinity of the delta and omicron variants. One explanation is that the S protein mutations increased the proportion of hydrophobic amino acids, such as leucine and phenylalanine, which may affect the binding affinity. Nevertheless, the interactions between AD08043 and different the RBDs of the variants of SARS-CoV-2 are all located near the interface of RBD-ACE2, indicating that AD08043 may have a broader anti-SARS-CoV-2 potential. The higher stability of AD08043 than PPS and (IdoA2S-GlcNS6S)_4_, and the favorable profile of its druggability further highlights the advantages of AD08043.

## 4. Materials and Methods

### 4.1. Preparation of the Pentasaccharide Library

**Figure 9:**
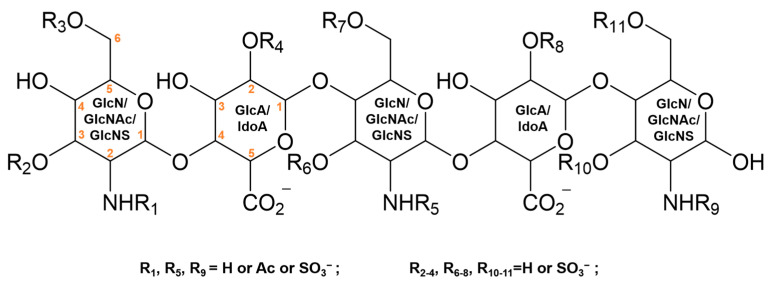
Illustration of the pentasaccharide structures in the library. The carbon atom serial number is shown in the monosaccharide plane structure.

The library (in .sdf format), containing 3D structures of HS-mimetics pentasaccharide sequences, was established in Chem3D 17.0. To mimic HS structure, the library contains pentasaccharide sequences of GlcN-HexUA-GlcN-HexUA-GlcN with possible sulfate substitutions (Figure 9). The configurations of the pentasaccharides were obtained through optimizing each structure to minimize the molecules’ energy, using the DISCOVERY STUDIO v4.5 computer program [27]. The energy minimization was performed using CHARMm force fields until an RMS gradient of 0.1 kcal/(mol × Å) was reached.

### 4.2. Virtual Screening

For the virtual screening validation test, the crystal structure of the SARS-CoV-2 RBD (PDB ID: 6VSB) was used. The protein was protonated at a physiological pH and was then converted to .pdbqt from the .pdb format using AutoDock MGLTools [28]. The pentasaccharides in the library were prepared using the built-in script ‘prepare_ligand4.py’ in MGLTools to assign polar hydrogen atoms and convert the format from .pdb to .pdbqt. The binding interface of the SARS-CoV-2 RBD as the target of the screening was predicted via Schrodinger SiteMap [29]. In the rescoring procedure, the following amino acids at the binding interface were allowed to be flexible: Arg346, Phe347, Ala348, Ser349, Ala352, Trp353, Asn354, Arg355, Lys356, Arg357, Lys444, Asn448, Asn450, Tyr451, Arg466, and Arg509, which is also consistent with previous work [4]. The box size was set to 60.00 Å × 60.00 Å × 60.00 Å with Glide [30,31] as the receptor-based virtual screening program.

### 4.3. Molecular Docking

The molecular docking studies were performed utilizing Autodock VINA in YASARA [32,33]. Binding affinities between the pentasaccharides with several variants’ RBDs, including B.1.1.7 (alpha) variant (PDB ID:7FEM), B.1.351 (beta) variant (PDB ID:7VX1), B.1.617.2 (delta) variant (PDB ID:7W92), P.1(gamma) variant (PDB ID:7SBT), and B.1.1.529 (omicron BA.1) variant (PDB ID:7XO4), were investigated. To remove bumps and ascertain the covalent geometry of the oligosaccharide ligands, the RBD structures were all energy-minimized via the NOVA force field [34]. The blind dockings were undertaken by defining simulation cell boxes of 60.00 Å × 45.00 Å × 55.00 Å for all variants of the SARS-CoV-2 RBD. The docking studies were carried out through the built-in docking simulation macro ‘dock_run.mcr’ using AMBER03 force field [35] with 25 poses and 9 clusters for each situation.

### 4.4. Molecular Dynamics

To examine whether the fluctuation and conformational changes of the model constructed from protein–ligand docking are reliable, a molecular dynamics (MD) simulation of the binding between the RBD of SARS-CoV-2 S protein and the selected pentasaccharide was performed. The AMBER14 force field was utilized for the MD simulation, as implemented in the YASARA program. For the long-range coulomb forces beyond the 8 Å cutoff, the MD simulation used periodic boundary conditions and the particle-mesh Ewald method. NaCl was used at a concentration of 0.9%, and the density of HOH was 0.997 g/mL in the MD cell. No restraints were applied during the MD simulation and the settings employed in the second equilibration dynamics were used. The energies and coordinates were saved every 100 ps with a total simulation length of 80 ns at constant temperature (298 K) and uncontrolled pressure in an NVT ensemble. The structural stability of the RBD-pentasaccharide complex was examined by analyzing the average values of potential energy with root mean square deviation (RMSD) throughout the trajectory. The RMSD profiles of the MD structures (Figures S2 and S3) show that the variation of the RMSD values tends to be stable, which assumes that the structures at equilibrium have been obtained and the last MD structure was chosen for the analysis.

The molecular mechanics Poisson–Boltzmann surface area (MM-PBSA) was used to compute the binding free energies of the complexes [36]. The calculations were conducted using the YASARA program. The cumulative free energy of the binding is calculated as follows:

ΔE_MMPBSA_ = G_complex_ − (G_protein_ + G_ligand_) (1)

where G_complex_ is the total MMPBSA energy of the protein–ligand complex, and G_protein_ and G_ligand_ are the highest occupied molecular orbital energies of the separated protein and ligands.

### 4.5. Visualization of Protein–Ligand Complex

LigPlot analysis was used to study the protein–ligand interactions by automatically generating schematic 2D representations of the protein–ligand complexes. The results are shown in a simple and informative diagram of the intermolecular interactions and their strengths, including hydrogen bonds, hydrophobic interactions, and atom accessibilities. The electrostatic potential map of the SARS-CoV-2 S protein’s RBD was generated from the crystal structure (PDB: 6VSB) and visualized using PyMoL (version 2.5.4 by Schrödinger).

### 4.6. Quantum Chemical Analysis

The electronic analysis of the selected pentasaccharide and other oligosaccharides was carried out using GAUSSIAN 09 [37], while the orbitals were shown using Multiwfn [38] and VMD 1.9.3 [39] software. The gradient-corrected (density functional theory) DFT with the three-parameter hybrid functional (B3) [40] for the exchange part, and the Lee–Yang–Parr (LYP) correlation function [41], was utilized to compute the molecular structure, vibrational frequencies, and energies of the optimized structures [42]. Moreover, to further explain the dispersion interactions that the B3LYP function is unable to describe, B3LYP-D3 was employed [43]. Meanwhile, the basis set 6-311 + G(d,p) was augmented by polarization functions on heavy atoms, polarization functions on hydrogen atoms, and diffuse functions for both hydrogen and heavy atoms [44]. The optimized geometries have been used to calculate the HOMO and LUMO energy parameters in this study.

Δ*E_g_* = E_LUMO_ − E_HOMO_ (2)

where Δ*E_g_* is the energy difference between the calculated excited state (LUMO) and the ground state (HOMO), E_LUMO_ is the lowest unoccupied molecular orbital energy, and E_HOMO_ is the highest occupied molecular orbital energy.

### 4.7. ADMET Study

The potential druggability of the selected pentasaccharides was evaluated using the web-based platform ADMETlab2.0 (https://admetmesh.scbdd.com/service/evaluation/index (accessed on 20 July 2023)). ADMETlab2.0 [45] utilizes a series of high-quality prediction models trained via a multi-task graph attention framework to conveniently and efficiently implement the calculation and prediction of the physicochemical properties, pharmacokinetics, and toxicology of the ligands. Molecules were uploaded in SMILES format. The web server automatically standardizes the input SMILES strings and computes all the endpoints including physicochemical properties, medicinal chemistry friendliness, and the ADMET (absorption, distribution, metabolism, excretion, and toxicity) properties.

## 5. Conclusions

The multidimensional biological functions of HS offer a wide range of pharmacological development potentials through targeting HS–protein interactions. Although hundreds of HS-binding proteins have been identified, knowledge of the exact structures of HS binding to a defined protein is still limited. One primary challenge in development of HS-mimetics is to identify a specific HS structure that interacts with a target protein with high affinity. A typical example is the heparin pentasaccharide binding to antithrombin [46]. With the rapid progress in the illustration of protein structures, virtual screening provides a powerful tool for the preliminary identification of HS structures. As such, computer-aided study is an efficient and economical way to rationally design drug candidates, and our work provides an example, showing the potential of this approach. This established procedure may be applied to a broad-spectrum screening of oligosaccharides–protein interactions, to explore potential novel drug candidates.

Despite the high throughput and efficiency of virtual screening, the most challenging task in the development of oligo/polysaccharide drugs is production of the compounds. The successful synthesis of the anticoagulant fondaparinux [47] demonstrates the possibility of chemically synthesizing oligosaccharides; however, the complicated chemical process including protection, activation, coupling, and de-protection, requires multiple steps, often with low yield. Nevertheless, the emerging synthetic biology is expected to break the bottle-necks of organic synthesis, making the synthesis of complicated carbohydrate structures possible [48], adding another pathway to the preparation of HS-mimetics for pharmaceutical development purposes. Yet, when considering the highly complicated structures of oligo/polysaccharides, more efficient synthesis procedures will be required, with tremendous effort needed to meet the requirement of diverse oligosaccharides structures.

## Figures and Tables

**Table 1:** Results of the molecular docking between SARS-CoV-2 RBD and the ligands.

Ligand Name	Binding Free Energies (kcal/mol)	Dissociation Constant (μM)
AD08043	−7.149	5.751
(IdoA2S-GlcNS6S)_4_	−6.797	10.417
PPS	−6.247	26.357

**Table 2:** Detailed information on the non-bond forces between SARS-CoV-2 RBD and the oligosaccharides.

Ligand Name	Hydrogen Bond Receptor Atom—Ligand Atom (Bond Length)	Hydrophobic Contact
AD08043	(1) Arg355:NH1—O15 (3.18 Å); (2) Arg355:N—O20 (3.06 Å); (3) Arg466:NH1—O17 (3.14 Å); (4) Arg466:NH2—O16 (3.11 Å); (5) Arg466:NH2—O16 (3.18 Å); (6) Tyr396:OH—O28 (2.94 Å); (7) Tyr396:OH—O30 (3.14 Å); (8) Leu517:N—O4 (3.11 Å).	(1) Trp353; (2) Asn354; (3) Pro426; (4) Asp428; (5) Phe429; (6) Phe464; (7) Phe515; (8) Glu516; (9) His519.
(IdoA2S-GlcNS6S)_4_	(1) Asn334:ND2—O7 (2.94 Å); (2) Asn334:ND2—O12 (3.12 Å); (3) Arg355:N—O73 (3.11 Å); (4) Arg357:N—O62 (3.19 Å); (5) Arg357:NE—O42 (2.95 Å); (6) Arg357:NE—O53 (3.06 Å); (7) Arg357:NH1—O57 (3.00 Å); (8) Arg357:NH1—O89 (2.96 Å); (9) Arg357:NH2—O42 (3.24 Å); (10) Asn360:O—O9 (2.99 Å); (11) Tyr396:OH—2O12 (2.94 Å); (12) Arg466:NH1—O92 (3.14 Å); (13) Arg466:NH2—N76 (3.18 Å).	(1) Pro337; (2) Trp353; (3) Asn354; (4) Ser359; (5) Pro426; (6) Pro463; (7) Phe464; (8) Glu465; (9) Ser514; (10) Phe515; (11) Glu516.
PPS	(1) Arg355:NH1—O14 (2.93 Å); (2) Arg355:NH1—O33 (3.14 Å); (3) Arg357:NH1—O5 (3.03 Å); (4) Arg357:NH2—O55 (3.35 Å); (5) Arg466:NH1—O17 (3.28 Å); (6) Arg466:NH2—O23 (3.03 Å).	(1) Tyr396; (2) Pro426; (3) Asp428; (4) Lys462; (5) Pro463; (6) Phe464; (7) Glu465; (8) Ser514; (9) Phe515; (10) Leu517; (11) His519.

**Table 3:** Detailed information on non-bond forces between AD08043 and RBDs of different variants SARS-CoV-2.

Receptor Name	Hydrogen Bond Receptor Atom—Ligand Atom (Bond Length)	Hydrophobic Contact
B.1.1.7 (alpha) variant RBD	(1) Arg355:NH1—O10 (3.18 Å); (2) Arg355:NH2—O9 (3.33 Å); (3) Arg355:NH2—O10 (3.23 Å); (4) Lys462:NZ—O (3.20 Å); (5) Arg466:NH2—O16 (2.84 Å); (6) Arg466:NH2—O25 (2.95 Å).	(1) Tyr396; (2) Pro426; (3) Asp428; (4) Phe429; (5) Thr430; (6) Pro463; (7) Phe464; (8) Ser514; (9) Glu516.
B.1.351 (beta) variant RBD	(1) Arg355:NH1—O30 (3.22 Å); (2) Thr430:N—O7 (3.01 Å); (3) Arg466:NE—O20 (3.28 Å); (4) Arg466:N—O26 (3.06 Å); (5) Ile468:N—O21 (2.80 Å); (6) Phe515:O—N (2.91 Å).	(1) Asp428; (2) Phe429; (3) Phe464; (4) Glu465; (5) Asp467; (6) Glu516; (7) Leu517.
P.1 (gamma) variant RBD	(1) Asn448:ND2—O22 (2.73 Å); (2) Arg509:NH2—O26 (2.96 Å).	(1) Asn343; (2) Ala344; (3) Thr345; (4) Arg346; (5) Ser373; (6) Trp436; (7) Asn437; (8) Asn440; (9) Leu441; (10) Asp442; (11) Asn450; (12) Tyr451.
B.1.617.2 (delta) variant RBD	(1) Arg346:NH2—O5 (2.80 Å); (2) Arg452:NH2—O29 (2.89 Å); (3) Thr470:OG1—O13 (3.13 Å); (4) Thr470:OG1—O15 (2.96 Å); (5) Thr470:O—O20 (2.74 Å); (6) Gly482:O—N1 (3.26 Å).	(1) Ala348; (2) Tyr351; (3) Ala352; (4) Asn354; (5) Tyr451; (6) Ile468; (7) Glu471; (8) Ile472; (9) Phe490.
B.1.1.529 (omicron BA.1) variant RBD	(1) Asn343:O—O8 (2.98 Å); (2) Thr345:N—O9 (3.16 Å); (3) Arg346:N—O9 (2.81 Å); (4) Ser349:OG—O20 (3.06 Å); (5) Asn354:ND2—O (2.96 Å); (6) Asn450:ND2—O25 (3.01 Å).	(1) Glu340; (2) Ala344; (3) Phe347; (4) Ala348; (5) Tyr351; (6) Ala352; (7) Tyr449; (8) Leu452.

**Table 4:** Detailed information on the non-bond forces of SARS-CoV-2 RBD and AD08043 at different time points during the MD simulation period.

Time Point	Hydrogen Bond Receptor Atom—Ligand Atom (Bond Length)	Hydrophobic Contact
0 ns	(1) Arg355:NH1—O10 (3.07 Å); (2) Arg355:O—O20 (2.65 Å); (3) Arg466:NH1—O16 (2.77 Å); (4) Arg466:NH2—O16 (2.83 Å); (5) Phe515:O—O4 (2.66 Å).	(1) Trp353; (2) Tyr396; (3) Asp428; (4) Phe464; (5) Ser514; (6) Glu516; (7) Leu517; (8) His519.
10 ns	(1) Asn394:ND2—O32 (3.07 Å); (2) His519:ND1—O10 (3.01 Å).	/
20 ns	(1) Arg357:NH1—O16 (2.60 Å); (2) Arg357:NH2—O15 (2.81 Å); (3) Arg357:NH2—O16 (2.88 Å); (4) Asn360:ND2—O31 (2.91 Å); (5) Tyr396:OH—O22 (2.73 Å).	/
30 ns	(1) Arg357:NH1—O16 (2.67 Å); (2) Arg357:NH2—O15 (2.76 Å); (3) Asn360:ND2—O29 (2.80 Å); (4) Tyr396:OH—O25 (3.02 Å).	/
40 ns	/	/
50 ns	(1) Tyr396:OH—O30 (2.80 Å).	(1) Arg355; (2) Arg357.
60 ns	(1) Arg357:NH1—O17 (2.81 Å); (2) Arg357:NH2—O15 (2.96 Å); (3) Arg357:NH2—O17 (3.28 Å); (4) Asn360:ND2—O15 (3.23 Å); (5) Asn394:ND2—O29 (2.70 Å).	(1) Ace332; (2) Thr393.
70 ns	(1) Asn354:OD1—O4 (3.10 Å); (2) Arg357:N—O29 (3.04 Å).	(1) Arg346; (2) Phe347; (3) Ala348; (4) Arg355; (5) Lys356.
80 ns	(1) Arg346:NH2—O7 (2.92 Å);(2) Lys356:NZ—O13 (3.03 Å); (3) Arg357:N—O29 (2.97 Å).	(1) Phe347; (2) Ala348.

**Table 5:** FMOs results of AD08043, PPS, and (IdoA2S-GlcNS6S)_4_.

Ligand Name	HOMO (eV)	LUMO (eV)	∆*E_g_* = E_LUMO_ − E_HOMO_ (eV)
AD08043	−6.660	−0.088	6.572
PPS	−6.256	0.027	6.283
(IdoA2S-GlcNS6S)4	−6.034	0.058	6.092

**Table 6:** Predicted ADMET properties of AD08043, PPS, and (IdoA2S-GlcNS6S)_4_.

Parameters	AD08043	PPS	(IdoA2S-GlcNS6S)_4_
No. of H bond acceptors	36	50	81
No. of H bond donors	18	10	30
Topological Polar Surface area, TPSA ([Å]^2^)	568.400	720.460	1264.350
Lipophilicity, log P	−6.291	−8.807	−15.092
Water Solubility, log S	1.948	5.717	8.771
Pfizer Rule	Accepted	Accepted	Accepted
Pgp inhibitor	No	No	No
Plasma Protein Binding (PPB)	3.180%	61.866%	52.737%
Volume Distribution (VD) (L/kg)	0.140	−0.324	−0.926
Blood–Brain Barrier (BBB) Penetration	No	No	No
CYP1A2 inhibitor	No	No	No
CYP2C19 inhibitor	No	No	No
CYP2C9 inhibitor	No	No	No
CYP2D6 inhibitor	No	No	No
CYP3A4 inhibitor	No	No	No
Human hepatotoxicity (H-HT)	Negative	Positive	Positive
Rat Oral Acute Toxicity	Low toxicity	High toxicity	Low toxicity
Skin Sensitization	Negative	Positive	Negative
Carcinogenicity	Negative	Positive	Negative
Respiratory Toxicity	Negative	Positive	Positive
Drug-likeness	Yes	No	No

## Data Availability

All data are available in the manuscript and Supplementary Materials.

## Supplementary Materials

The following supporting information can be downloaded at https://www.mdpi.com/article/10.3390/ijms242216115/s1.
