# Supplementary material for: Identification of a Pentasaccharide Lead Compound with High Affinity to the SARS-CoV-2 Spike Protein via In Silico Screening
Source: Int J Mol Sci. 2023 Nov 9;24(22):16115. doi: 10.3390/ijms242216115 (PMC10671481; doi:10.3390/ijms242216115)
Supplement: Supplementary file 1 [file ijms-24-16115-s001.zip › ijms-2661619-supplementary.pdf]

Supplementary Materials

Identification of a pentasaccharide lead compound with high affinity to the SARS-CoV-2 spike protein by in silico screening

Binjie Li [1], Tianji Zhang [2], Hui Cao [3], Vito Ferro [4], Jingping Li [1,5,*] and Mingjia Yu [6,*]

1 Beijing Advanced Innovation Center for Soft Matter Science and Engineering, Beijing University of Chemical Technology, Beijing, China; 2023400367@buct.edu.cn
2 Division of Chemistry and Analytical Science, National Institute of Metrology, Beijing, China; zhangtianji@nim.ac.cn
3 College of Life Science and Technology, Beijing University of Chemical Technology, Beijing, China; caohui@mail.buct.edu.cn
4 School of Chemistry & Molecular Biosciences, The University of Queensland, Brisbane, Australia; v.ferro@uq.edu.au.
5 Department of Medical Biochemistry and Microbiology, Uppsala University, Uppsala, Sweden;
6 School of Chemistry and Chemical Engineering, Beijing Institute of Technology, Beijing, China;

* Correspondence: 2018880006@buct.edu.cn (J.-P.L.) and 6120210204@bit.edu.cn (M.Y.);

Table S1. The binding affinity between top10 pentasaccharides and the RBD.

Ligand name	Structure	Bind.energy (-kcal/mol)
AD08043	GlcNS-β-(1→4)-GlcA-α-(1→4)-GlcNAc3S-β-(1→4)-IdoA-α-(1→4)-GlcNAc	7.149
AD04615	GlcN3S--β-(1→4)-IdoA-α-(1→4)-GlcNAc6S--β-(1→4)-IdoA-α-(1→4)-GlcNAc	6.759
AD02532	GlcN6S-β-(1→4)-GlcA-β-(1→4)-GlcNAc3S-β-(1→4)-GlcA-α-(1→4)-GlcN6S	7.013
AD12490	GlcNAc-β-(1→4)-GlcA-α-(1→4)-GlcN3S-β-(1→4)-IdoA-α-(1→4)-GlcNAC	7.116
AD12596	GlcNAc-α-(1→4)-IdoA-α-(1→4)-GlcN3S-α-(1→4)-IdoA-α-(1→4)-GlcNAc6S	7.092
AD00091	GlcN-β-(1→4)-IdoA-α-(1→4)-GlcNAc3S-β-(1→4)-IdoA-β-(1→4)-GlcNS	6.867
AD00337	GlcN-β-(1→4)-GlcA-α-(1→4)-GlcNS-α-(1→4)-GlcA-α-(1→4)-GlcN6S	6.862
AD00652	GlcN-β-(1→4)-IdoA2S-β-(1→4)-GlcNAc-β-(1→4)-GlcA-β-(1→4)-GlcNS	6.840
AD02344	GlcN6S-α-(1→4)-GlcA-α-(1→4)-GlcN6S-β-(1→4)-IdoA-β-(1→4)-GlcNAc	7.010
AD03123	GlcN6S-β-(1→4)-IdoA2S-β-(1→4)-GlcN3S-α-(1→4)-IdoA-α-(1→4)-GlcNAc	6.730

Table S2. The binding affinity between AD08043 and the RBD of variants of concern.

Target protein	Bind.energy (-kcal/mol)
Origin	7.149
Gama (P.1)	7.089
Alpha (B.1.1.7)	6.818
Beta (B.1.351)	6.508
Omicron_BA.1 (B.1.1.529)	6.507
Delta (B.1.617.2)	6.179

Table S3. The detailed information on non-bond forces of SARS-CoV-2 RBD and the top10 pentasaccharides, with the exception of AD08043.

Ligand Name	Hydrogen Bond Receptor atom—Ligand atom (bond length)	Hydrophobic Contact
AD04615	(1) Phe464:O—N2 (3.04 Å); (2) Arg466:N—O (3.28 Å); (3) Arg466:NH1—O12 (3.08 Å); (4) Arg466:NH1—O13 (3.28 Å); (5) Arg466:NH2—O13 (2.87 Å); (6) His519:NE2—O24 (3.00 Å);	(1) Trp353; (2) Arg355; (3) Tyr396; (4) Pro463; (5) Glu465; (6) Asp467; (7) Ile468; (8) Ser469; (9) Ser514; (10) Phe515; (11) Glu516;
AD02532	(1) Asn354:OD1—O9 (3.07 Å); (2) Arg355:NH1—C17 (2.84 Å); (3) Lys462:NHZ—O22 (3.05 Å); (4) Lys462:NHZ—O23 (3.20 Å); (5) Arg466:NH1—O1 (2.98 Å); (6) Arg466:NH1—O4 (3.11 Å); (7) Arg466:NH1—O6 (2.98 Å); (8) Arg466:NH1—O14 (3.22 Å); (9) Arg466:NH2—O14 (3.08 Å);	(1) Trp353; (2) Lys356; (3) Arg357; (4) Tyr396; (5) Pro426; (6) Pro463; (7) Phe464; (8) Glu465; (9) Ile468
AD12490	(1) Arg355:O—N (2.97 Å); (2) Tyr396:OH—O10 (3.13 Å); (3) Asp427:N—O20 (2.97 Å);	(1) Lys356; (2) Arg357; (3) Pro426; (4) Asp428; (5) Pro463; (6) Phe464; (7) Arg466; (8) Ser514; (9) His519;
AD12596	(1) Arg355:N—O7 (3.09 Å); (2) Arg357:NH1—O12 (2.99 Å); (3) Phe464:O—O9 (3.09 Å); (4) Arg466:NH1—O6 (3.10 Å); (5) Arg466:NH2—O4 (3.00 Å); (6) Arg466:NH2—O8 (3.22 Å);	(1) Trp353; (2) Asn354; (3) Tyr396; (4) Pro426; (5) Pro463; (6) Glu465; (7) Ser514; (8) Phe515; (9) Glu516; (10) His519
AD00091	(1) Asn354:OD1—N (3.22 Å); (2) Arg355:NH1—O17 (3.09 Å); (3) Arg466:NH1—O2 (2.75 Å);	(1) Ala348; (2) Ala352; (3) Trp353; (4) Arg357; (5) Tyr396; (6) Phe464; (7) Ile468; (8) Ser514; (9) Phe515; (10) Glu516; (11) His519;
AD00337	(1) Arg355:O—N1 (2.96 Å); (2) Arg357:NH1—O22 (3.00 Å); (3) Arg357:NH2—O22 (3.10 Å); (4) Arg357:NH2—O23 (3.06 Å); (5) Phe515:O—N (3.01 Å);	(1) Trp353; (2) Lys356; (3) Tyr396; (4) Pro426; (5) Phe429; (6) Thr430; (7) Phe464; (8) Glu465; (9) Arg466; (10) Ser514; (11) Glu516; (12) His519;
AD00652	(1) Arg355:O—N1 (2.96 Å); (2) Arg355:NH1—O31 (3.01 Å); (3) Arg357:NH1—O15 (2.97 Å); (4) Arg357:NH1—O28 (3.02 Å); (5) Arg357:NH2—O28 (3.16 Å); (6) Arg466:NH2—O25 (2.93 Å); (7) His519:NE2—O7 (3.08 Å);	(1) Asn354; (2) Lys356; (3) Tyr396; (4) Phe429; (5) Phe464; (6) Phe515; (7) Glu516; (8) Leu517;
AD02344	(1) Thr345:N—O26 (3.19 Å); (2) Arg355:NH1—O8 (2.94 Å); (3) Arg357:N—O14 (3.33 Å); (4) Arg357:NH1—O1 (2.90 Å); (5) Arg466:NH1—O10 (2.98 Å); (6) Arg466:NH2—O10 (3.17 Å);	(1) Ala344; (2) Arg346; (3) Trp353; (4) Asn354; (5) Tyr396; (6) Ser399; (7) Phe464;
AD03123	(1) Arg355:NH1—O13 (2.96 Å); (2) Arg357:NH1—O18 (3.20 Å); (3) His519:NE2—O10 (3.26 Å);	(1) Tyr396; (2) Pro426; (3) Asp428; (4) Phe429; (5) Pro463; (6) Phe464; (7) Arg466; (8) Glu516;

Table S4. The binding free energy between oligasaccharides with their respective protein target by MMP-BSA method.

Ligand name	Target protein	Bind.energy (-kcal/mol)
AD08043	SARS-CoV-2 RBD	74.496
$(IdoA2S\text{-}GlcNS6S)_4$	SARS-CoV-2 RBD	69.090
PPS	SARS-CoV-2 RBD	46.109
AD08043	SARS-CoV-2 Alpha variant RBD	49.728
AD08043	SARS-CoV-2 Beta variant RBD	106.836
AD08043	SARS-CoV-2 Gama variant RBD	81.110
AD08043	SARS-CoV-2 Delta variant RBD	8.044
AD08043	SARS-CoV-2 Omicron_BA.1 variant RBD	26.135

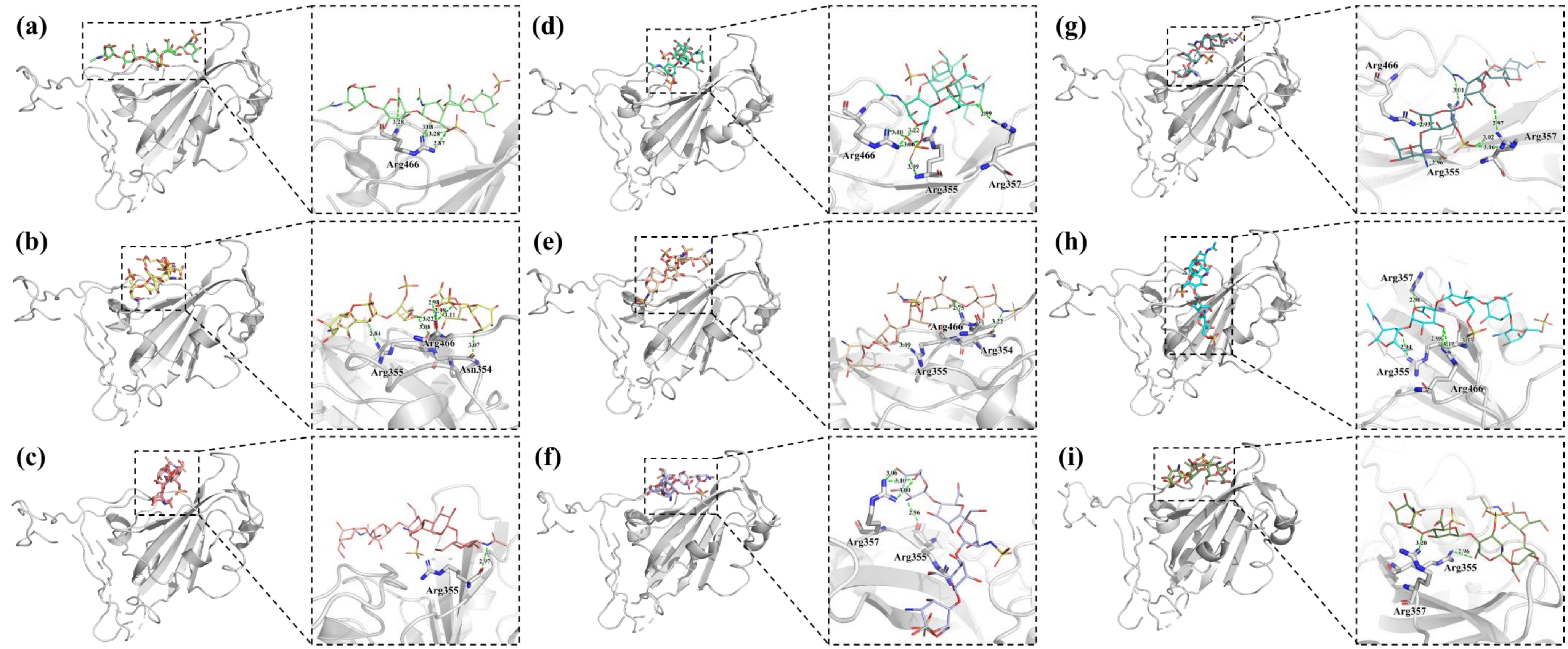

Figure S1. Interactions between the interface amino acids of SARS-CoV-2 RBD and top10 pentasaccharides, with the exception of AD08043. **(a)** AD04615; **(b)** AD02532; **(c)** AD12490; **(d)** AD12596; **(e)** AD00091; **(f)** AD00337; **(g)** AD00652; **(h)** AD02344; **(i)** AD03123.

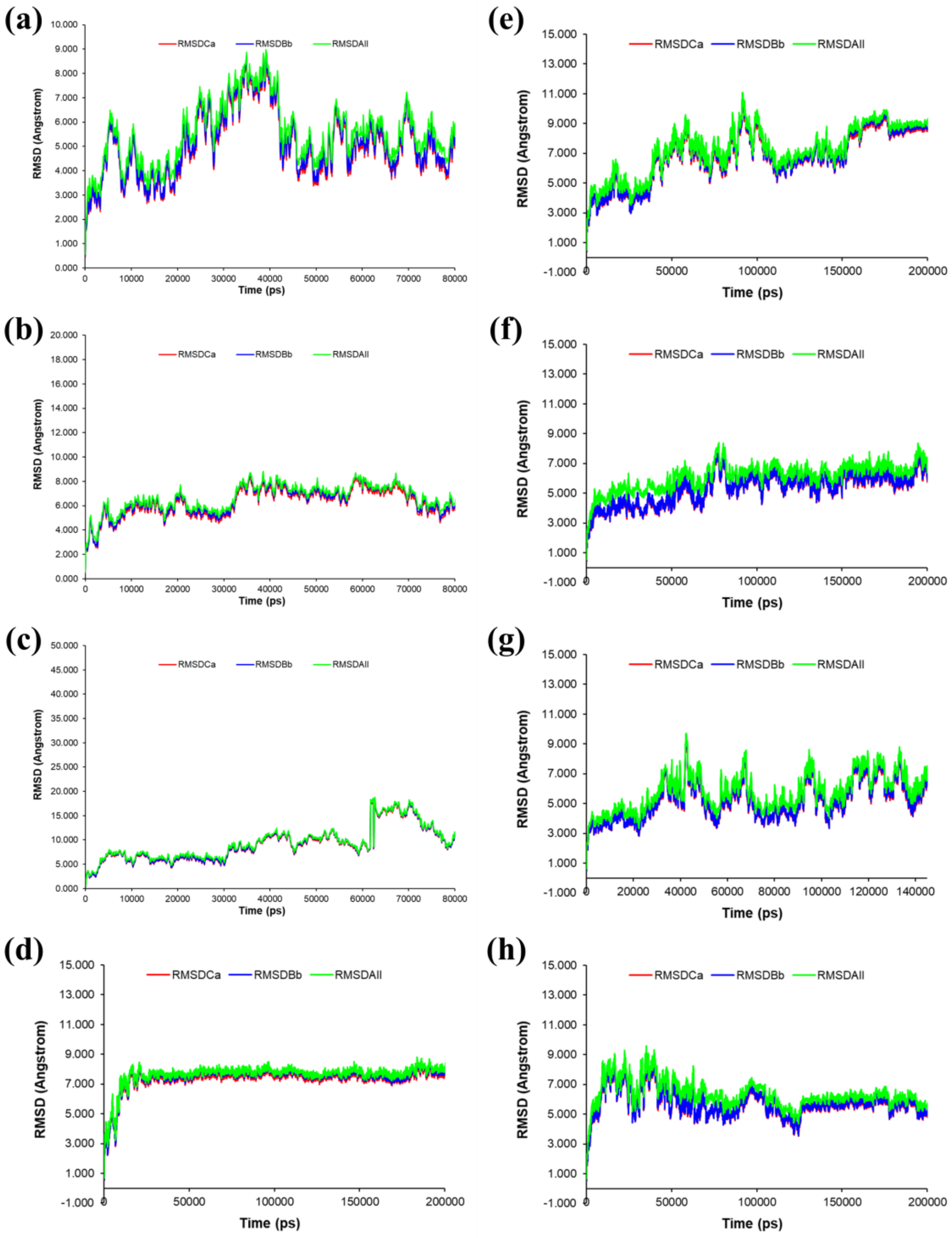

Figure S2. The RMSD profiles of MD simulations of the variants of SARS-CoV-2 RBD with oligosaccharides. **(a)** SARS-CoV-2 RBD-AD08043 (80 ns); **(b)** SARS-CoV-2 RBD-PPS (80 ns); **(c)** SARS-CoV-2 RBD-(IdoA2S-GlcNS6S)$_4$ (80 ns); **(d)** SARS-CoV-2 Alpha variant RBD-AD08043 (200 ns); **(e)** SARS-CoV-2 Beta variant RBD-AD08043 (200 ns); **(f)** SARS-CoV-2 Gamma variant RBD-AD08043 (200 ns); **(g)** SARS-CoV-2 Delta variant RBD-AD08043 (145 ns); **(h)** SARS-CoV-2 Omicron BA.1 variant RBD-AD08043 (200 ns). The RMSD values of Calpha [RMSDCa], backbone [RMSDBb] and all-heavy atom [RMSDAll] are shown in red, blue and green, respectively.

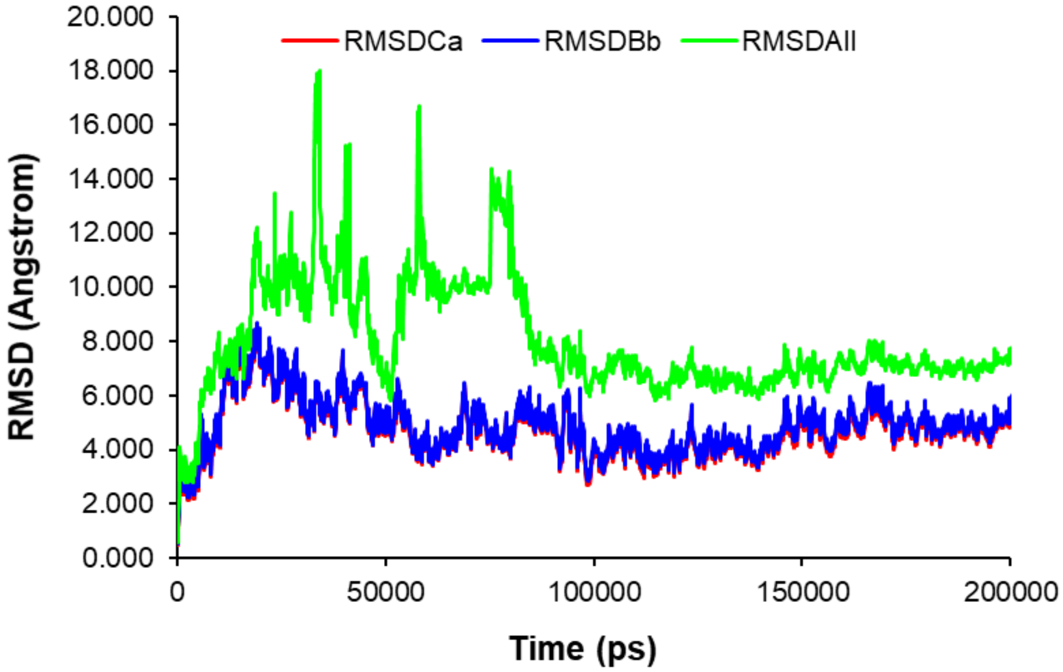

Figure S3. The RMSD profiles of 200 ns MD simulations of the point mutated SARS-CoV-2 S protein RBD (amino acids 346, 356, and 357 mutated to alanin) with AD08043. The RMSD values of Calpha [RMSDCa], backbone [RMSDBb] and all-heavy atom [RMSDAll] are shown in red, blue and green, respectively.

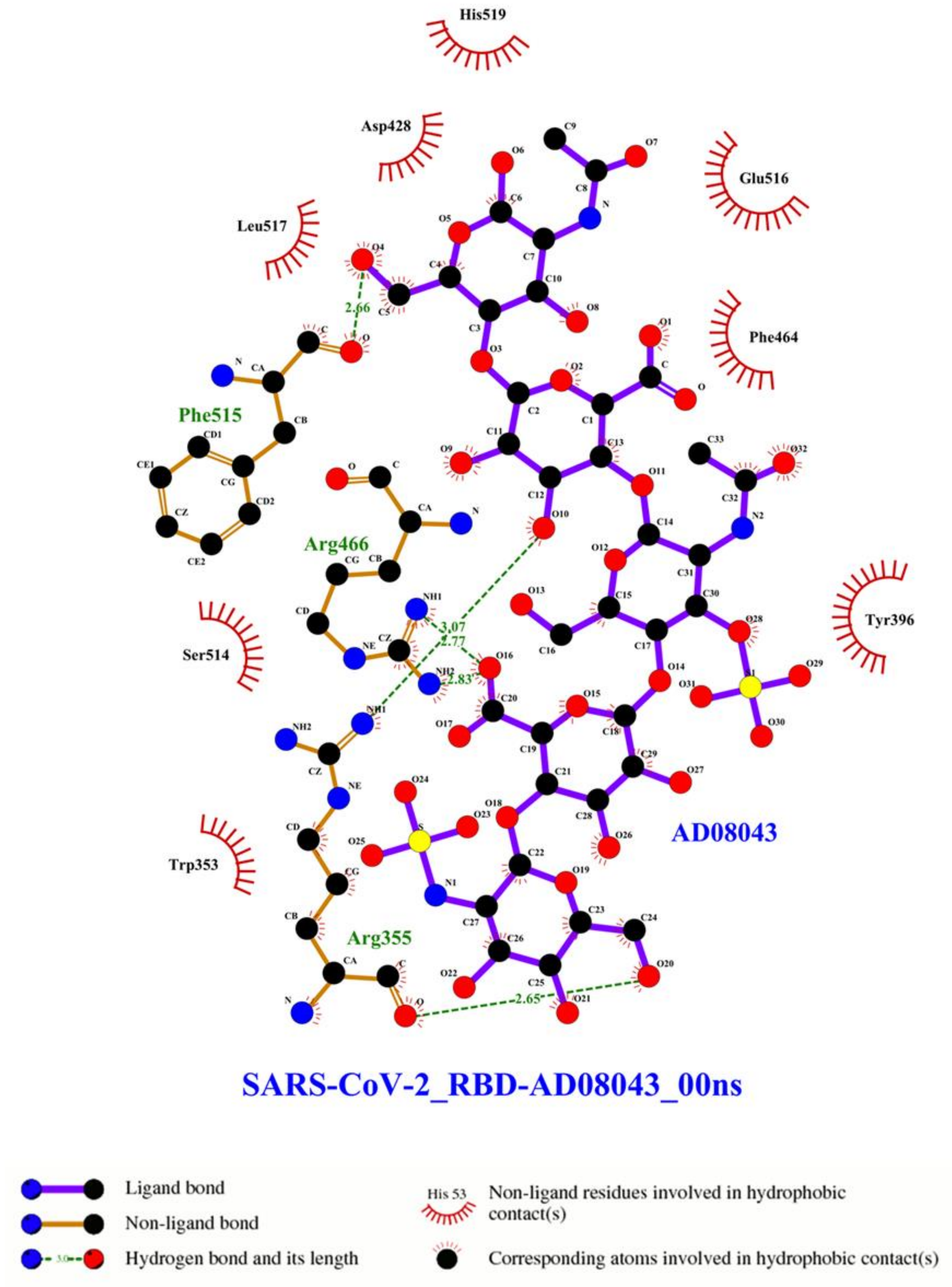

Figure S4. The 2D schematic diagrams of protein-ligand interactions between SARS-CoV-2 S protein RBD with AD08043 in the original state before MD simulations.

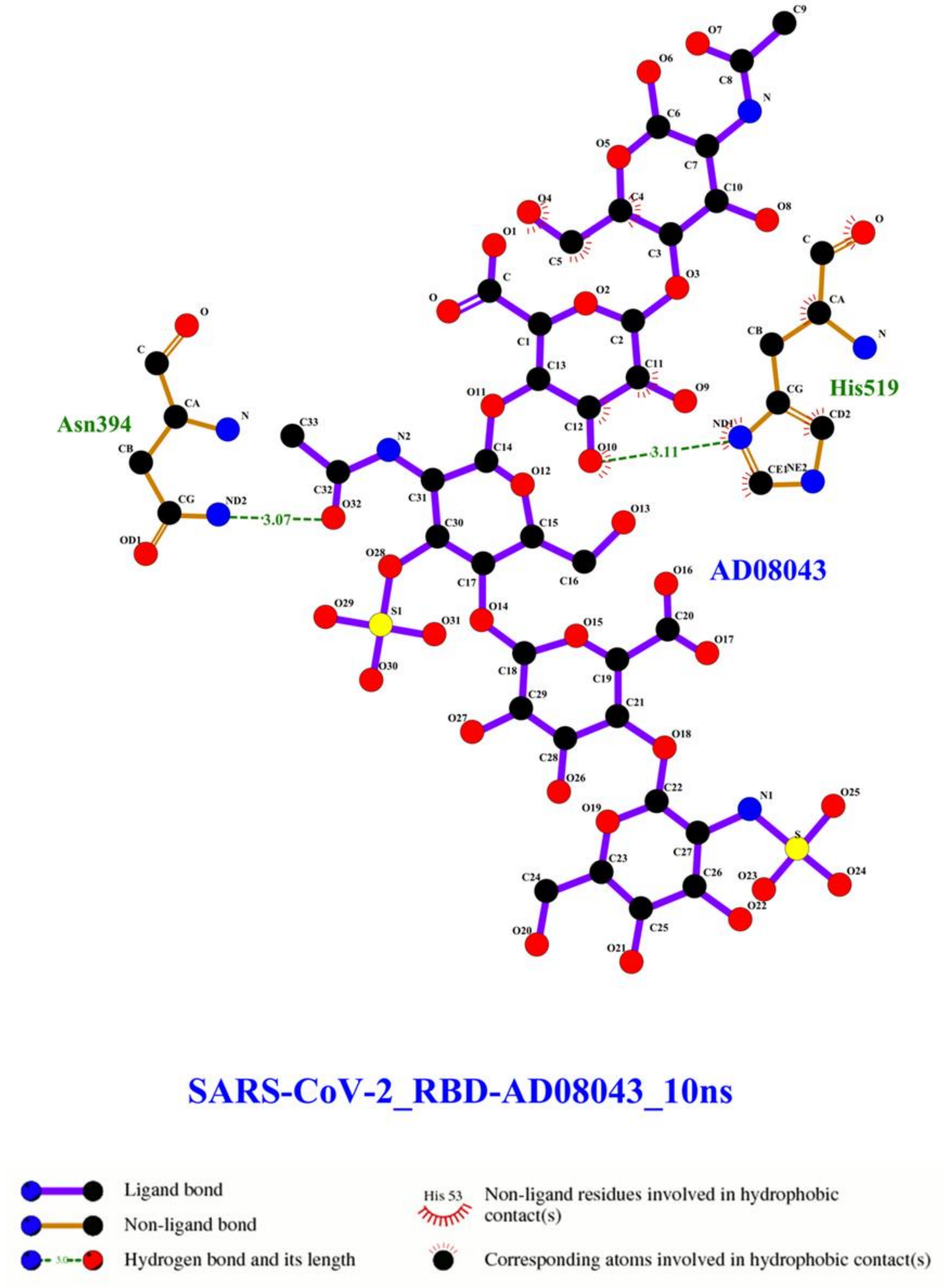

Figure S5. The 2D schematic diagrams of protein-ligand interactions between SARS-CoV-2 S protein RBD with AD08043 in the 10 ns during MD simulations.

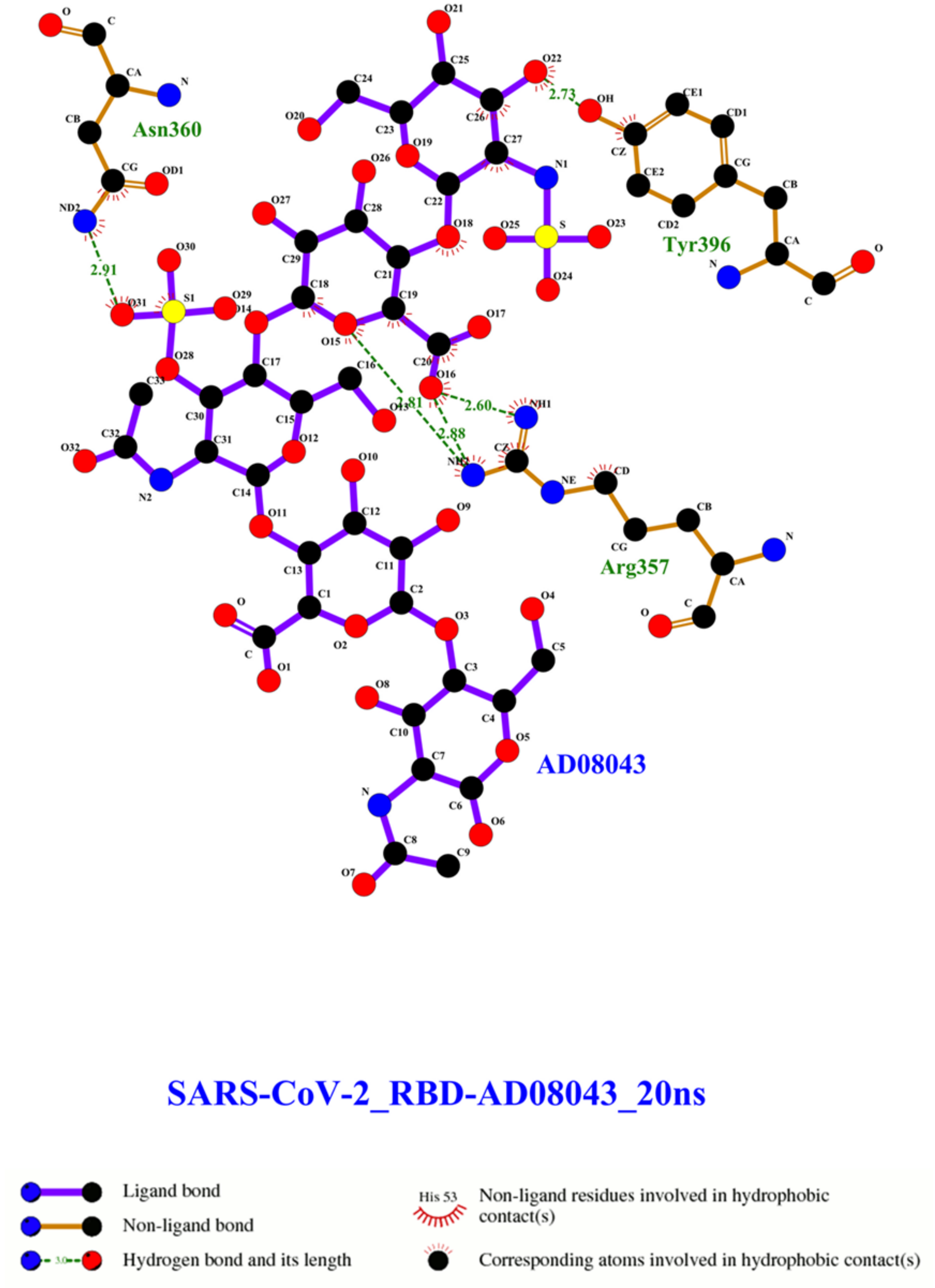

Figure S6. The 2D schematic diagrams of protein-ligand interactions between SARS-CoV-2 S protein RBD with AD08043 in the 20 ns during MD simulations.

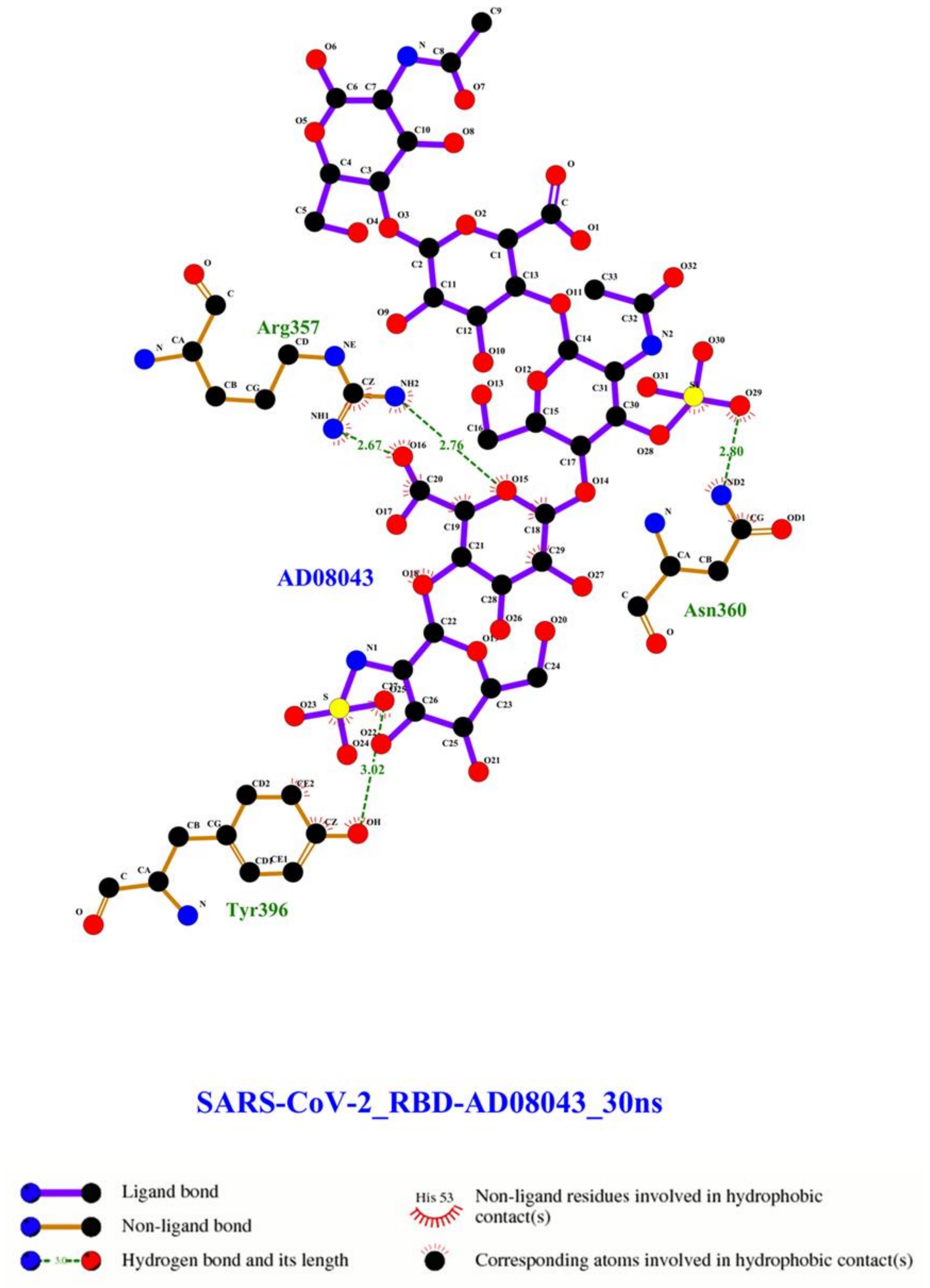

Figure S7. The 2D schematic diagrams of protein-ligand interactions between SARS-CoV-2 S protein RBD with AD08043 in the 30 ns during MD simulations.

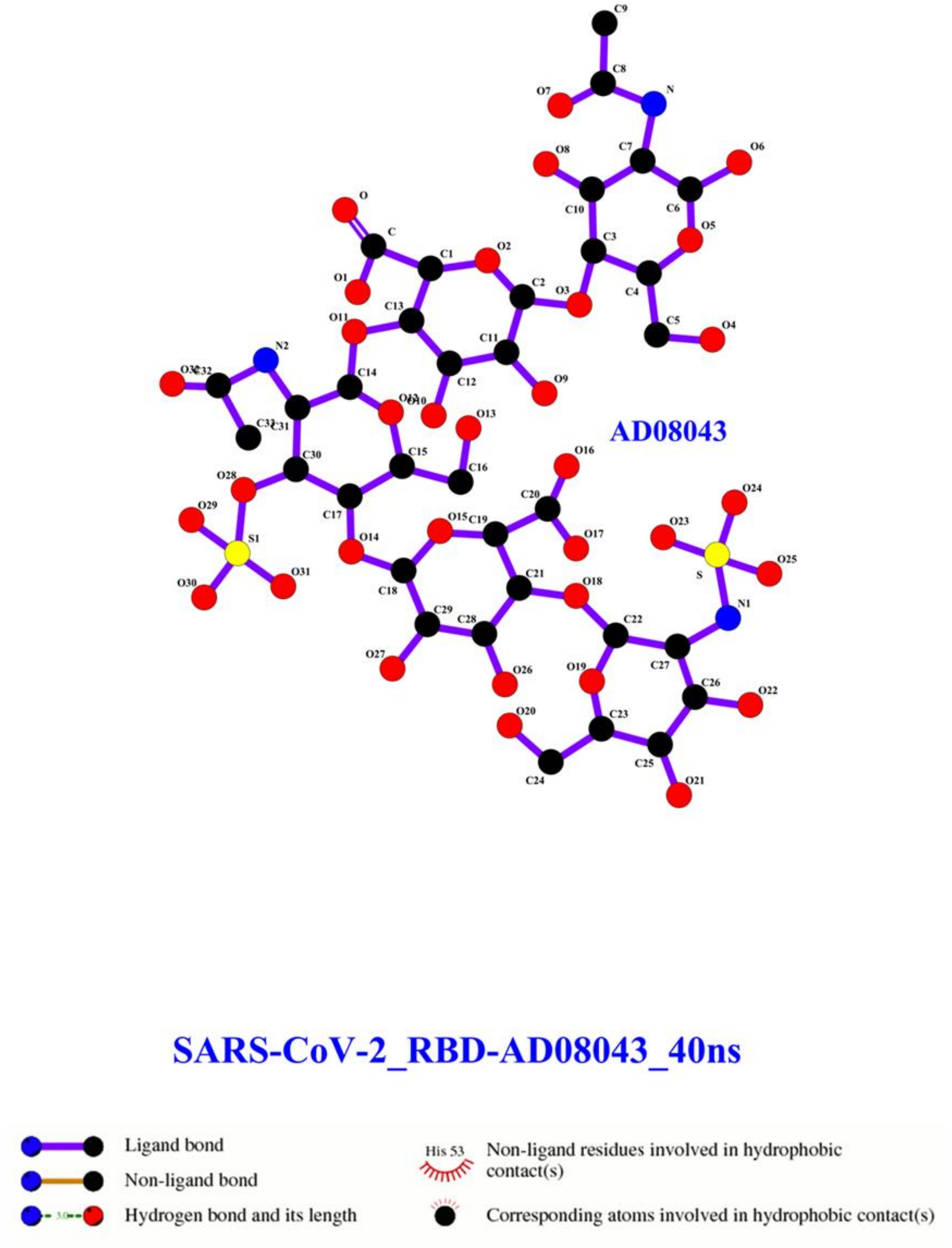

Figure S8. The 2D schematic diagrams of protein-ligand interactions between SARS-CoV-2 S protein RBD with AD08043 in the 40 ns during MD simulations.

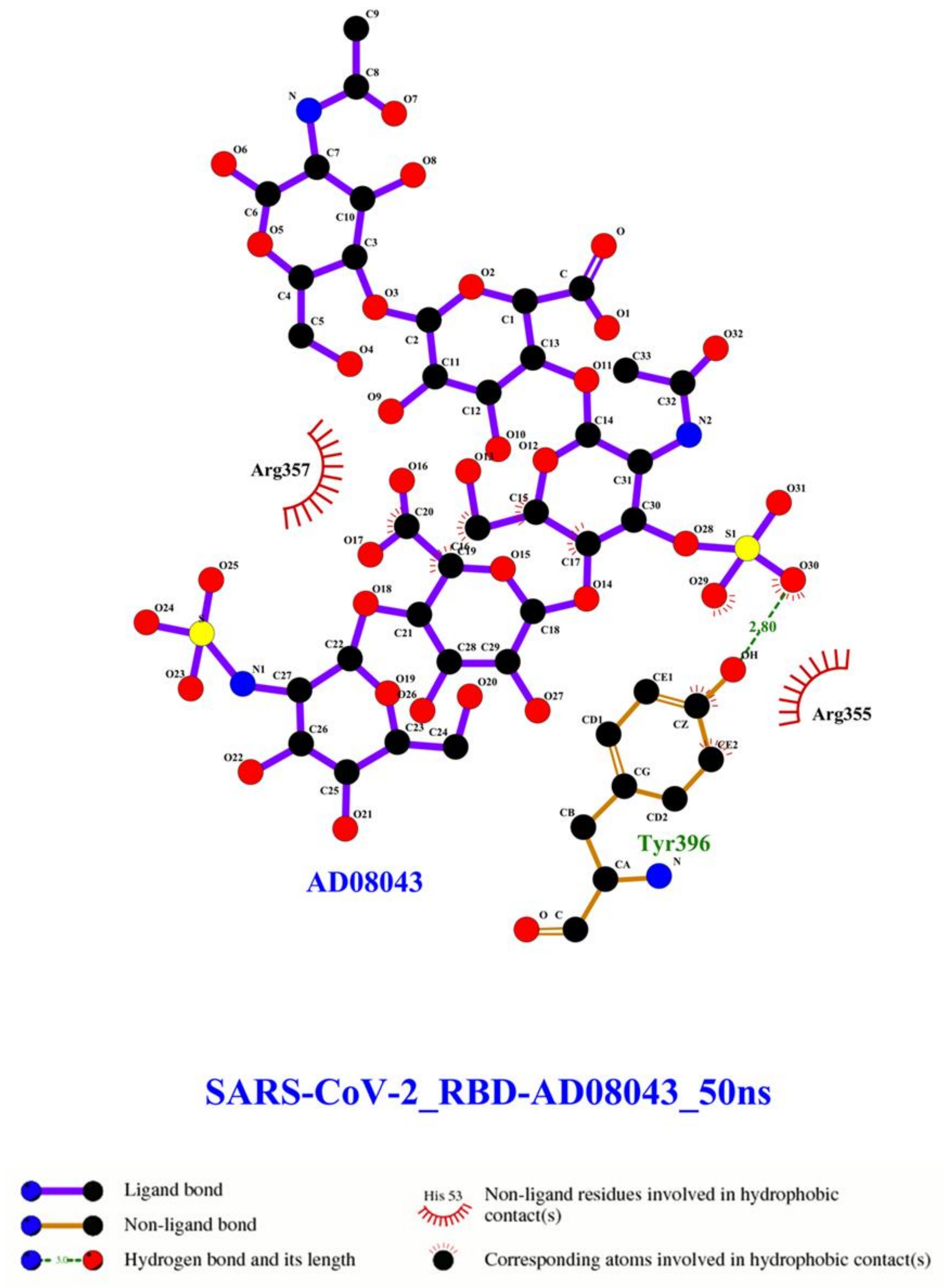

Figure S9. The 2D schematic diagrams of protein-ligand interactions between SARS-CoV-2 S protein RBD with AD08043 in the 50 ns during MD simulations.

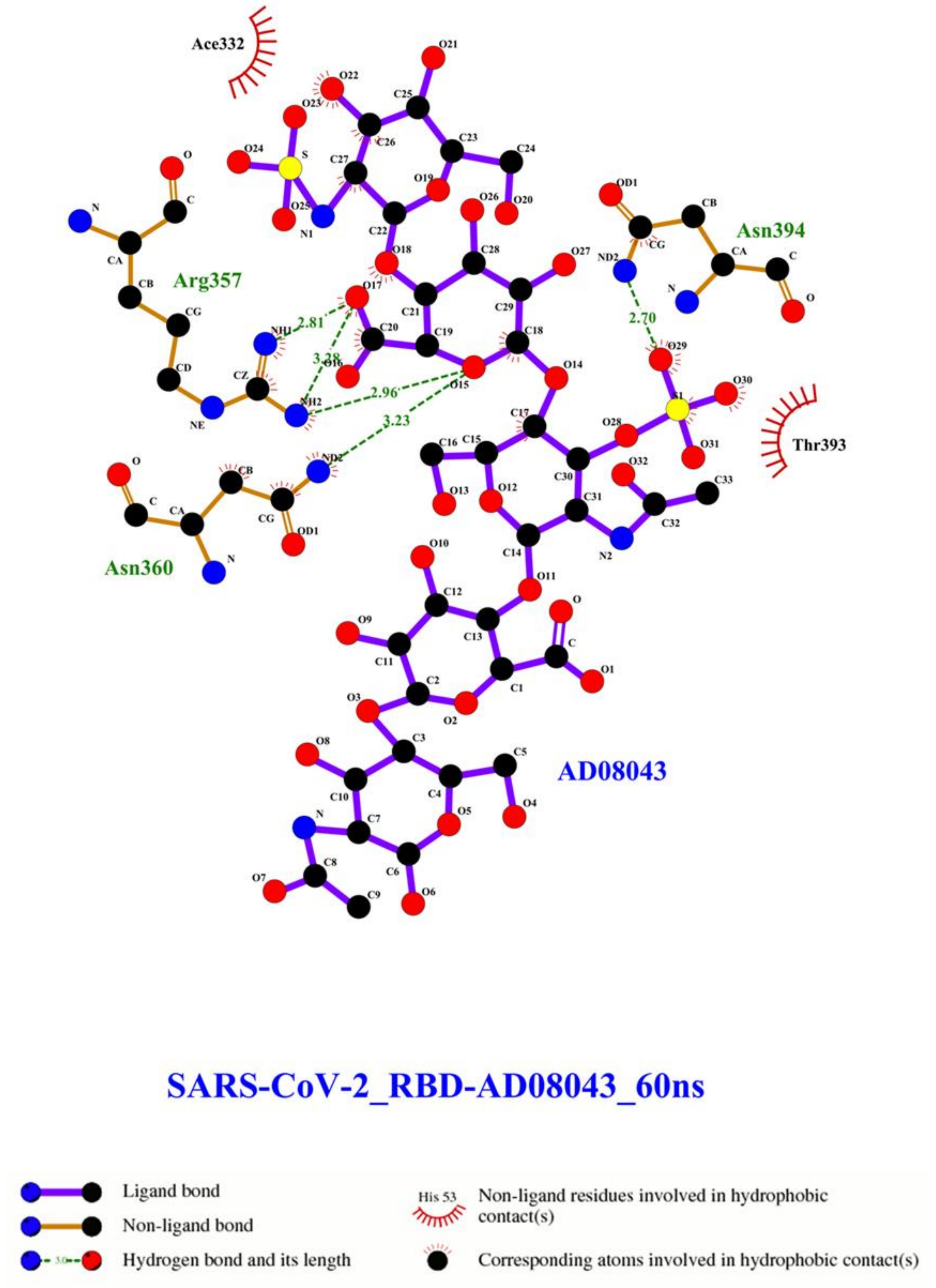

Figure S10. The 2D schematic diagrams of protein-ligand interactions between SARS-CoV-2 S protein RBD with AD08043 in the 60 ns during MD simulations.

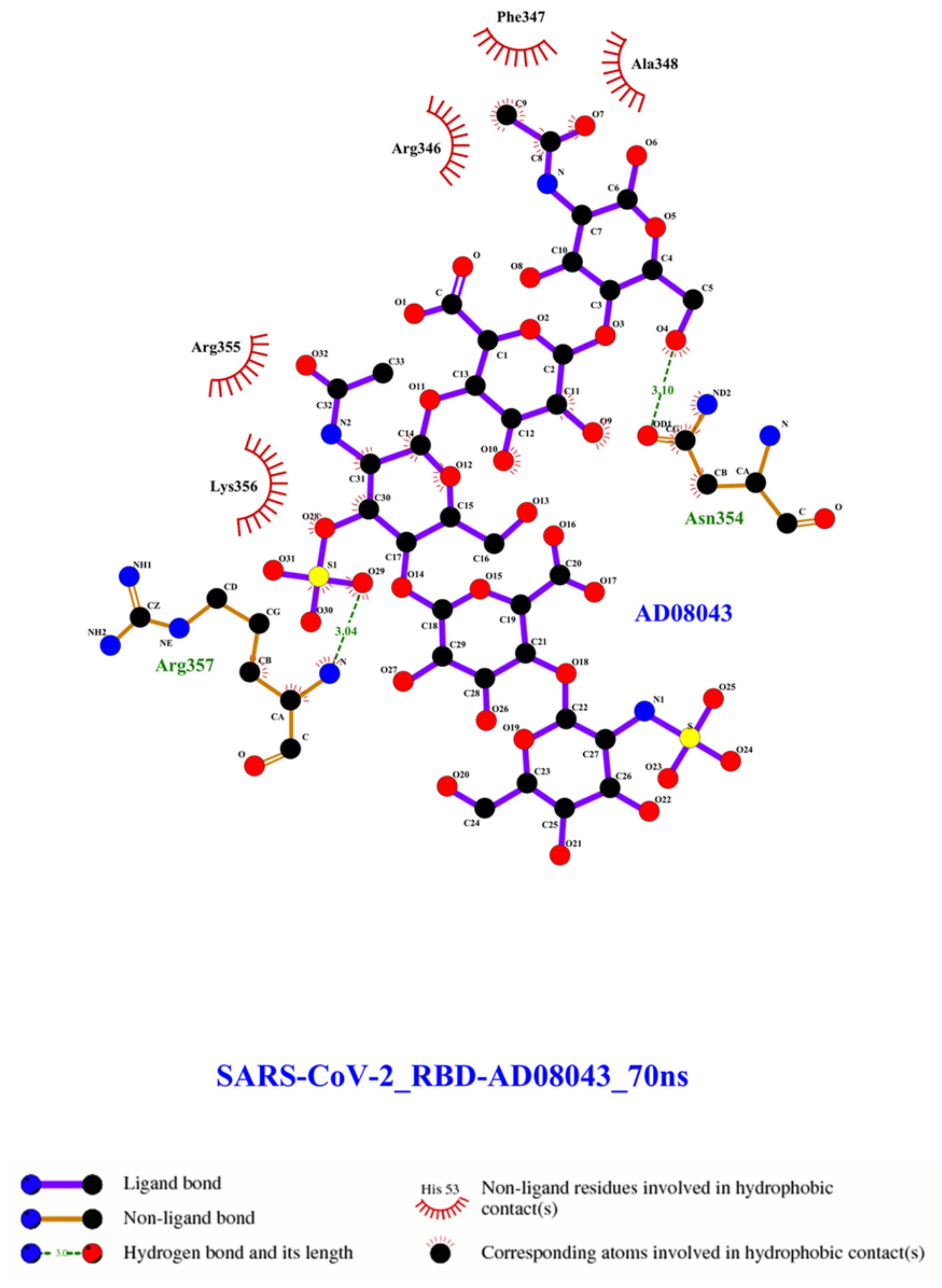

Figure S11. The 2D schematic diagrams of protein-ligand interactions between SARS-CoV-2 S protein RBD with AD08043 in the 70 ns during MD simulations.

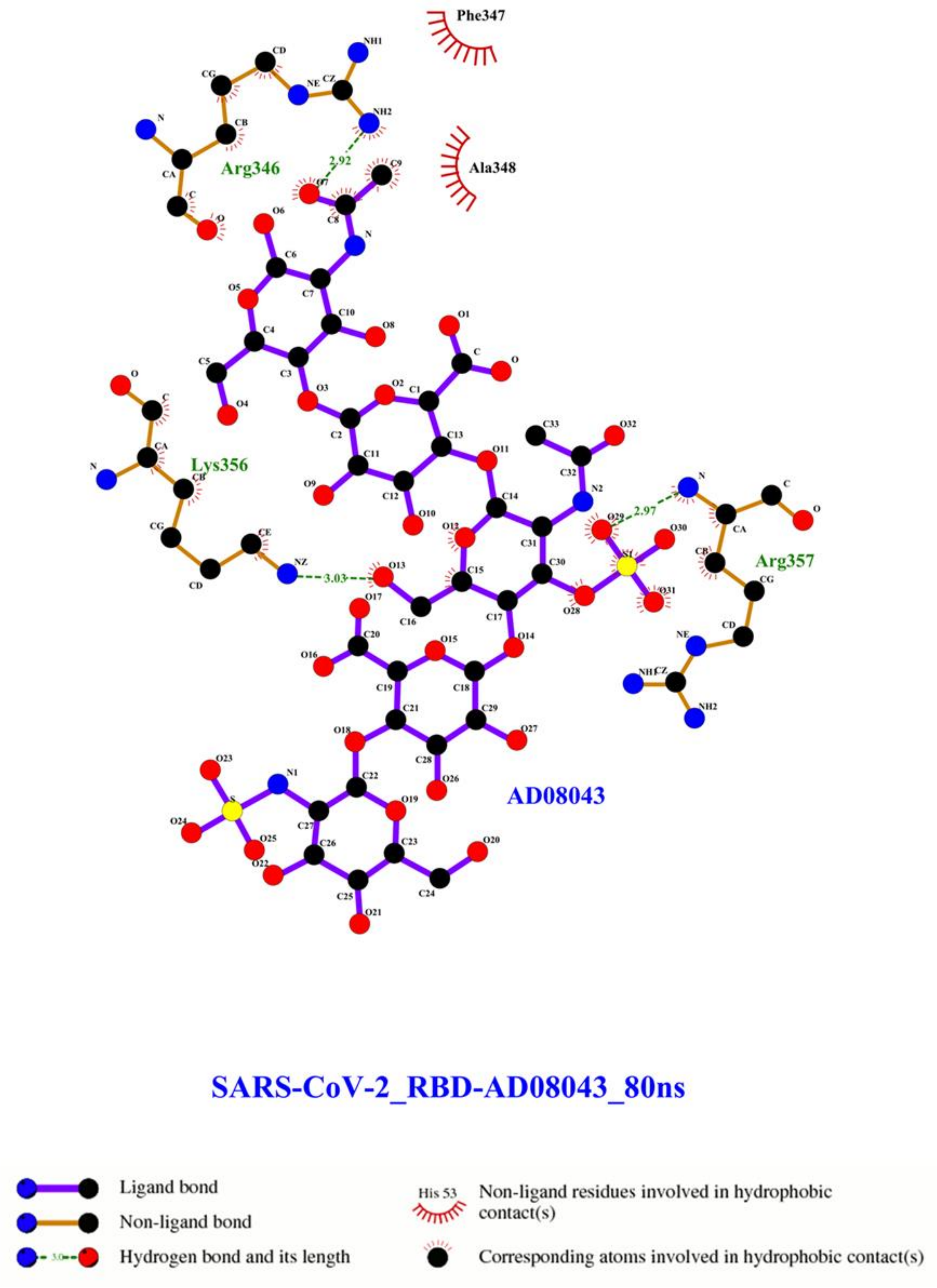

Figure S12. The 2D schematic diagrams of protein-ligand interactions between SARS-CoV-2 S protein RBD with AD08043 in the 80 ns during MD simulations.

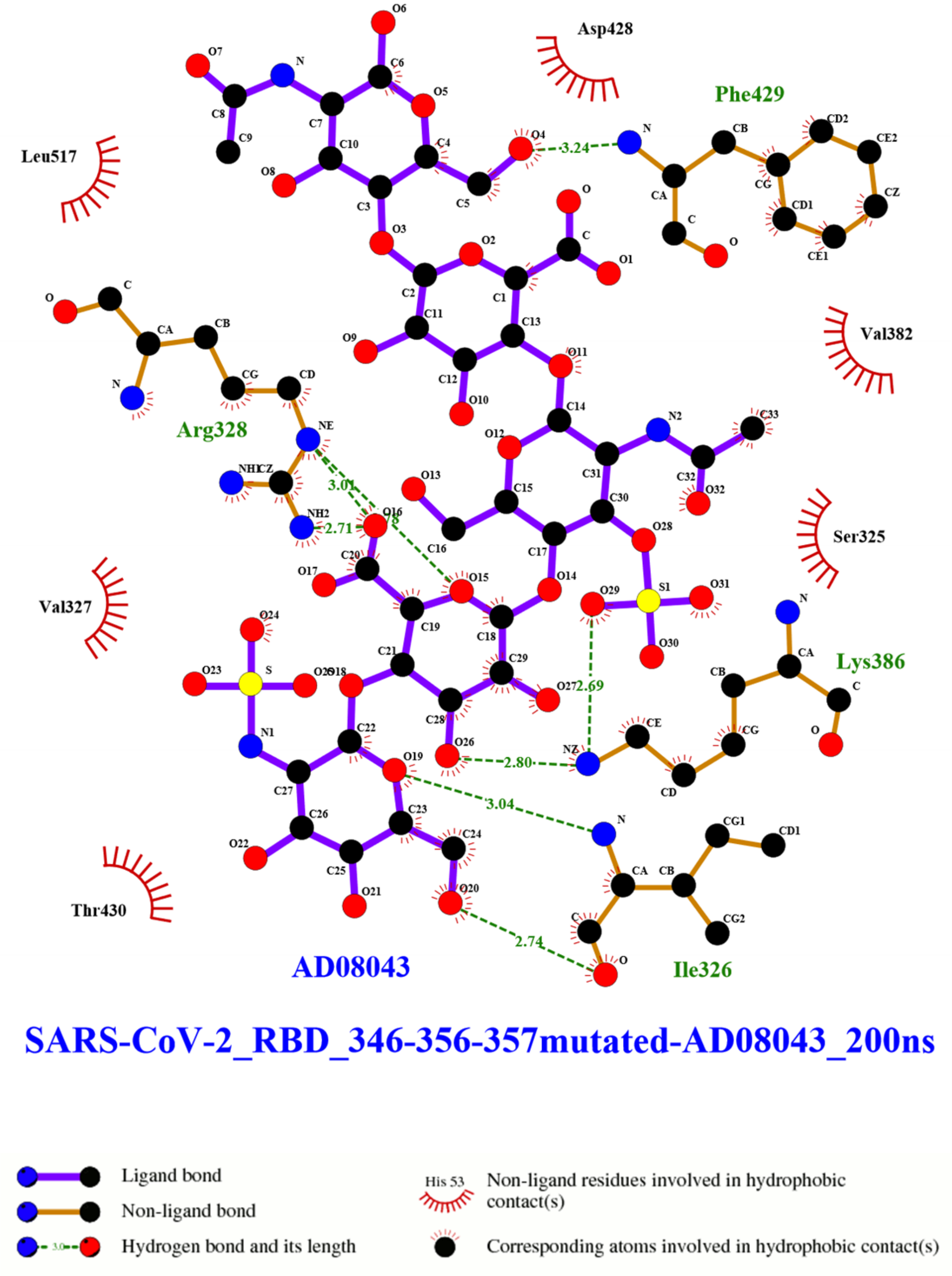

Figure S13. The 2D schematic diagrams of protein-ligand interactions between point mutated SARS-CoV-2 S protein RBD (amino acids 346, 356, and 357 mutated to alanin) with AD08043 in the 200 ns during MD simulations.
